# Pitfalls in assessing stromal tumor infiltrating lymphocytes (sTILs) in breast cancer

**DOI:** 10.1038/s41523-020-0156-0

**Published:** 2020-05-12

**Authors:** Zuzana Kos, Elvire Roblin, Rim S. Kim, Stefan Michiels, Brandon D. Gallas, Weijie Chen, Koen K. van de Vijver, Shom Goel, Sylvia Adams, Sandra Demaria, Giuseppe Viale, Torsten O. Nielsen, Sunil S. Badve, W. Fraser Symmans, Christos Sotiriou, David L. Rimm, Stephen Hewitt, Carsten Denkert, Sibylle Loibl, Stephen J. Luen, John M. S. Bartlett, Peter Savas, Giancarlo Pruneri, Deborah A. Dillon, Maggie Chon U. Cheang, Andrew Tutt, Jacqueline A. Hall, Marleen Kok, Hugo M. Horlings, Anant Madabhushi, Jeroen van der Laak, Francesco Ciompi, Anne-Vibeke Laenkholm, Enrique Bellolio, Tina Gruosso, Stephen B. Fox, Juan Carlos Araya, Giuseppe Floris, Jan Hudeček, Leonie Voorwerk, Andrew H. Beck, Jen Kerner, Denis Larsimont, Sabine Declercq, Gert Van den Eynden, Lajos Pusztai, Anna Ehinger, Wentao Yang, Khalid AbdulJabbar, Yinyin Yuan, Rajendra Singh, Crispin Hiley, Maise al Bakir, Alexander J. Lazar, Stephen Naber, Stephan Wienert, Miluska Castillo, Giuseppe Curigliano, Maria-Vittoria Dieci, Fabrice André, Charles Swanton, Jorge Reis-Filho, Joseph Sparano, Eva Balslev, I-Chun Chen, Elisabeth Ida Specht Stovgaard, Katherine Pogue-Geile, Kim R. M. Blenman, Frédérique Penault-Llorca, Stuart Schnitt, Sunil R. Lakhani, Anne Vincent-Salomon, Federico Rojo, Jeremy P. Braybrooke, Matthew G. Hanna, M. Teresa Soler-Monsó, Daniel Bethmann, Carlos A. Castaneda, Karen Willard-Gallo, Ashish Sharma, Huang-Chun Lien, Susan Fineberg, Jeppe Thagaard, Laura Comerma, Paula Gonzalez-Ericsson, Edi Brogi, Sherene Loi, Joel Saltz, Frederick Klaushen, Lee Cooper, Mohamed Amgad, David A. Moore, Roberto Salgado, the International Immuno-Oncology Biomarker Working Group, Aini Hyytiäinen, Akira I. Hida, Alastair Thompson, Alex Lefevre, Allen Gown, Amy Lo, Anna Sapino, Andre M. Moreira, Andrea Richardson, Andrea Vingiani, Andrew M. Bellizzi, Angel Guerrero, Anita Grigoriadis, Ana C. Garrido-Castro, Ashley Cimino-Mathews, Ashok Srinivasan, Balazs Acs, Baljit Singh, Benjamin Calhoun, Benjamin Haibe-Kans, Benjamin Solomon, Bibhusal Thapa, Brad H. Nelson, Carmen Ballesteroes-Merino, Carmen Criscitiello, Carolien Boeckx, Cecile Colpaert, Cecily Quinn, Chakra S. Chennubhotla, Cinzia Solinas, Damien Drubay, Dhanusha Sabanathan, Dieter Peeters, Dimitrios Zardavas, Doris Höflmayer, Douglas B. Johnson, E. Aubrey Thompson, Edith Perez, Ehab A. ElGabry, Elizabeth F. Blackley, Emily Reisenbichler, Ewa Chmielik, Fabien Gaire, Fang-I Lu, Farid Azmoudeh-Ardalan, Franklin Peale, Fred R. Hirsch, Gabriela Acosta-Haab, Gelareh Farshid, Glenn Broeckx, Harmut Koeppen, Harry R. Haynes, Heather McArthur, Heikki Joensuu, Helena Olofsson, Ian Cree, Iris Nederlof, Isabel Frahm, Iva Brcic, Jack Chan, James Ziai, Jane Brock, Jelle Weseling, Jennifer Giltnane, Jerome Lemonnier, Jiping Zha, Joana Ribeiro, Jochen K. Lennerz, Jodi M. Carter, Johan Hartman, Johannes Hainfellner, John Le Quesne, Jonathan W. Juco, Jose van den Berg, Joselyn Sanchez, Joël Cucherousset, Julien Adam, Justin M. Balko, Kai Saeger, Kalliopi Siziopikou, Karolina Sikorska, Karsten Weber, Keith E. Steele, Kenneth Emancipator, Khalid El Bairi, Kimberly H. Allison, Konstanty Korski, Laurence Buisseret, Leming Shi, Loes F. S. Kooreman, Luciana Molinero, M. Valeria Estrada, Maartje Van Seijen, Magali Lacroix-Triki, Manu M. Sebastian, Marcelo L. Balancin, Marie-Christine Mathieu, Mark van de Vijver, Marlon C. Rebelatto, Martine Piccart, Matthew P. Goetz, Matthias Preusser, Mehrnoush Khojasteh, Melinda E. Sanders, Meredith M. Regan, Michael Barnes, Michael Christie, Michael Misialek, Michail Ignatiadis, Michiel de Maaker, Mieke Van Bockstal, Nadia Harbeck, Nadine Tung, Nele Laudus, Nicolas Sirtaine, Nicole Burchardi, Nils Ternes, Nina Radosevic-Robin, Oleg Gluz, Oliver Grimm, Paolo Nuciforo, Paul Jank, Pawan Kirtani, Peter H. Watson, Peter Jelinic, Prudence A. Francis, Prudence A. Russell, Robert H. Pierce, Robert Hills, Roberto Leon-Ferre, Roland de Wind, Ruohong Shui, Samuel Leung, Sami Tabbarah, Sandra C. Souza, Sandra O’Toole, Sandra Swain, Sarah Dudgeon, Scooter Willis, Scott Ely, Shahinaz Bedri, Sheeba Irshad, Shiwei Liu, Shona Hendry, Simonetta Bianchi, Sofia Bragança, Soonmyung Paik, Sua Luz, Thomas Gevaert, Timothy d’Alfons, Tom John, Tomohagu Sugie, Uday Kurkure, Veerle Bossuyt, Venkata Manem, Vincente Peg Cámaea, Weida Tong, William T. Tran, Yihong Wang, Yves Allory, Zaheed Husain, Zsuzsanna Bago-Horvath

**Affiliations:** 1 Department of Pathology, BC Cancer - Vancouver, Vancouver, BC Canada; 2 Department of Biostatistics and Epidemiology, Gustave Roussy, University Paris-Saclay, Villejuif, France; 3 Oncostat U1018, Inserm, University Paris-Saclay, labeled Ligue Contre le Cancer, Villejuif, France; 4 National Surgical Adjuvant Breast and Bowel Project (NSABP)/NRG Oncology, Pittsburgh, PA USA; 5 Division of Imaging, Diagnostics, and Software Reliability (DIDSR); Office of Science and Engineering Laboratories (OSEL); Center for Devices and Radiological Health (CDRH), US Food and Drug Administration (US FDA), Silver Spring, MD USA; 6 Department of Pathology, University Hospital Antwerp, Antwerp, Belgium; 7 Department of Pathology, Ghent University Hospital, Cancer Research Institute Ghent (CRIG), Ghent, Belgium; 8 The Sir Peter MacCallum Cancer Centre, Melbourne, VIC Australia; 9 Peter MacCallum Department of Oncology, University of Melbourne, Melbourne, Victoria Australia; 10 Perlmutter Cancer Center, New York University Medical School, New York, NY USA; 11 Departments of Radiation Oncology and Pathology and Laboratory Medicine, Weill Cornell Medicine, New York, NY USA; 12 Department of Pathology, Istituto Europeo di Oncologia, University of Milan, Milan, Italy; 13 Department of Pathology and Laboratory Medicine, University of British Columbia, Vancouver, Canada; 14 Department of Pathology and Laboratory Medicine, Indiana University, Indianapolis, USA; 15 Department of Pathology, The University of Texas M.D. Anderson Cancer Center, Houston, TX USA; 16 Department of Medical Oncology, Institut Jules Bordet, Université Libre de Bruxelles, Brussels, Belgium; 17 Department of Pathology, Yale School of Medicine, New Haven, CT USA; 18 Laboratory of Pathology, National Cancer Institute, NIH, Bethesda, MD USA; 19 Institute of Pathology, Universitätsklinikum Gießen und Marburg GmbH, Standort Marburg and Philipps-Universität Marburg, Marburg, Germany; 20 German Breast Group, Neu-Isenburg, Germany; 21 Division of Research and Cancer Medicine, Peter MacCallum Cancer Centre, University of Melbourne, Melbourne, VIC Australia; 22 Ontario Institute for Cancer Research, Toronto, ON Canada; 23 University of Edinburgh Cancer Research Centre, Edinburgh, UK; 24 Department of Pathology, IRCCS Fondazione Instituto Nazionale Tumori and University of Milan, School of Medicine, Milan, Italy; 25 Department of Pathology, Brigham and Women’s Hospital, Boston, MA USA; 26 Department of Pathology, Dana Farber Cancer Institute, Boston, MA USA; 27 Institute of Cancer Research Clinical Trials and Statistics Unit, The Institute of Cancer Research, Surrey, UK; 28 Breast Cancer Now Toby Robins Research Centre, The Institute of Cancer Research, London, UK; 29 Vivactiv Ltd, Bellingdon, Bucks, UK; 30 Department of Medical Oncology and Division of Tumor Biology & Immunology, The Netherlands Cancer Institute, Amsterdam, The Netherlands; 31 Division of Molecular Pathology, The Netherlands Cancer Institute, Amsterdam, The Netherlands; 32 Department of Biomedical Engineering, Case Western Reserve University, Cleveland, OH USA; 33 Louis Stokes Cleveland Veterans Affairs Medical Center, Cleveland, OH USA; 34 Computational Pathology Group, Department of Pathology, Radboud University Medical Center, Nijmegen, Netherlands; 35 Department of Surgical Pathology Zealand University Hospital, Køge, Denmark; 36 Departamento de Anatomía Patológica, Universidad de La Frontera, Temuco, Chile; 37 Forbius, Montreal, QC Canada; 38 Department of Pathology, Peter MacCallum Cancer Centre Department of Pathology, Melbourne, VIC Australia; 39 Department of Pathology, Universidad de la Frontera, Temuco, Chile; 40 KU Leuven- Univerisity of Leuven, Department of Imaging and Pathology, Laboratory of Translational Cell & Tissue Research and KU Leuven- University Hospitals Leuven, Department of Pathology, Leuven, Belgium; 41 Department of Research IT, The Netherlands Cancer Institute, Amsterdam, The Netherlands; 42 Division of Tumor Biology & Immunology, The Netherlands Cancer Institute, Amsterdam, The Netherlands; 43 PathAI, Inc, Boston, MA USA; 44 Department of Pathology, Jules Bordet Institute, Brussels, Belgium; 45 Department of Pathology, GZA-ZNA, Antwerp, Belgium; 46 Department of Internal Medicine, Section of Medical Oncology, Yale Cancer Center, Yale School of Medicine, New Haven, CT USA; 47 Department of Clinical Genetics and Pathology, Skåne University Hospital, Lund University, Lund, Sweden; 48 Department of Pathology, Fudan University Shanghai Cancer Centre, Shanghai, China; 49 Centre for Evolution and Cancer; Division of Molecular Pathology, The Institute of Cancer Research, London, UK; 50 Icahn School of Medicine at Mt. Sinai, New York, NY 10029 USA; 51 Cancer Research UK Lung Cancer Centre of Excellence, University College London Cancer Institute, University College London, London, UK; 52 Departments of Pathology, Genomic Medicine, Dermatology, and Translational Molecular Pathology, The University of Texas MD Anderson Cancer Center, Houston, TX USA; 53 Department of Pathology and Laboratory Medicine, Tufts Medical Center, Boston, USA; 54 Charité - Universitätsmedizin Berlin, corporate member of Freie Universität Berlin, Humboldt-Universität zu Berlin, and Berlin Institute of Health, Institute of Pathology, Charitéplatz 1, 10117 Berlin, Germany; 55 Department of Medical Oncology and Research, Instituto Nacional de Enfermedades Neoplasicas, Lima, 15038 Peru; 56 University of Milan, Istituto Europeo di Oncologia, IRCCS, Milan, Italy; 57 Medical Oncology 2, Istituto Oncologico Veneto IOV - IRCCS, Padova, Italy; 58 Department of Surgery, Oncology and Gastroenterology, University of Padova, Padova, Italy; 59 Department of Medical Oncology, Institut Gustave Roussy, Villejuif, France; 60 Francis Crick Institute, Midland Road, London, UK; 61 Department of Pathology, Memorial Sloan Kettering Cancer Center, New York, NY USA; 62 Human Oncology and Pathogenesis Program, Memorial Sloan Kettering Cancer Center, New York, NY USA; 63 Montefiore Medical Center, Albert Einstein College of Medicine, Bronx, NY USA; 64 Department of Pathology, Herlev and Gentofte Hospital, Herlev, Denmark; 65 Department of Oncology, National Taiwan University Cancer Center, Taipei, Taiwan; 66 Department of Oncology, National Taiwan University Hospital, Taipei, Taiwan; 67 Graduate Institute of Oncology, College of Medicine, National Taiwan University, Taipei, Taiwan; 68 Centre de Lutte contre le cancer - Centre Jean Perrin, Clermont-Ferrand, France; 69 The University of Queensland Centre for Clinical Research and Pathology Queensland, Brisbane, QLD Australia; 70 Institut Curie, Paris Sciences Lettres Université, Inserm U934, Department of Pathology, Paris, France; 71 Pathology Department, Instituto de Investigación Sanitaria Fundación Jiménez Díaz (IIS-FJD) - CIBERONC, Madrid, Spain; 72 GEICAM-Spanish Breast Cancer Research Group, Madrid, Spain; 73 Nuffield Department of Population Health, University of Oxford, Oxford and Department of Medical Oncology, University Hospitals Bristol NHS Foundation Trust, Bristol, UK; 74 Department of Pathology, Bellvitge University Hospital, IDIBELL. Breast Unit. Catalan Institut of Oncology. L ‘Hospitalet del Llobregat’, Barcelona, 08908 Catalonia Spain; 75 University Hospital Halle (Saale), Institute of Pathology, Halle (Saale), Germany; 76 Molecular Immunology Unit, Institut Jules Bordet, Universitè Libre de Bruxelles, Brussels, Belgium; 77 Department of Biomedical Informatics, Emory University, Atlanta, GA USA; 78 Department of Pathology, National Taiwan University Hospital, Taipei, Taiwan; 79 Department of Pathology, Montefiore Medical Center and the Albert Einstein College of Medicine, Bronx, NY USA; 80 DTU Compute, Department of Applied Mathematics, Technical University of Denmark; Visiopharm A/S, Hørsholm, Denmark; 81 Pathology Department, Hospital del Mar, Parc de Salut Mar, Barcelona, Spain; 82 Breast Cancer Program, Vanderbilt-Ingram Cancer Center, Vanderbilt University Medical Center, Nashville, TN USA; 83 Biomedical Informatics Department, Stony Brook University, Stony Brook, NY USA; 84 Institute of Pathology, Charité Universitätsmedizin Berlin, Berlin, Germany; 85 Department of Pathology, Northwestern University Feinberg School of Medicine, Chicago, IL USA; 86 Department of Biomedical Informatics, Emory University School of Medicine, Atlanta, GA USA; 87 Department of Pathology, UCL Cancer Institute, UCL, London, UK; 88 University College Hospitals NHS Trust, London, UK; 89 Department of Oral and Maxillofacial Diseases, Helsinki, Finland; 90 Department of Pathology, Matsuyama Shimin Hospital, Matsuyama, Japan; 91 Surgical Oncology, Baylor College of Medicine, Houston, TX USA; 92 Roche Diagnostics, Machelen, Belgium; 93 PhenoPath Laboratories, Seattle, WA USA; 94 Research Pathology, Genentech Inc., South San Francisco, CA USA; 95 Department of Medical Sciences, University of Torino, Italy and Candiolo Cancer Institute - FPO, IRCCS, Candiolo, Italy; 96 Department of Pathology, New York University Langone Health, Center for Biospecimen Research and Development, New York, NY USA; 97 Department of Pathology, Johns Hopkins Hospital, Baltimore, MD USA; 98 Department of Pathology, Insituto Nazionale dei Tumori, Milan, Italy; 99 Department of Pathology, University of Iowa Hospitals and Clinics, Iowa City, IA USA; 100 Department of Oncology, IVO Valencia, Valencia, Spain; 101 Cancer Bioinformatics Lab, Cancer Centre at Guy’s Hospital, London, UK; 102 School of Life Sciences and Medicine, King’s College London, London, UK; 103 Dana-Farber Cancer Institute, Boston, MA USA; 104 Harvard Medical School, Boston, MA USA; 105 Departments of Pathology and Oncology, The Johns Hopkins Hospital, Baltimore, MD USA; 106 Department of Pathology, Karolinska Institute, Solna, Sweden; 107 Department of Pathology, New York University Langone Medical Centre, New York, NY USA; 108 Department of Pathology and Laboratory Medicine, UNC School of Medicine, Chapel Hill, NC USA; 109 Bioinformatics and Computational Genomics Laboratory, Princess Margaret Cancer Center, Toronto, ON Canada; 110 Department of Medicine, University of Melbourne, Parkville, VIC Australia; 111 Trev & Joyce Deeley Research Centre, British Columbia Cancer Agency, Victoria, BC Canada; 112 Providence Cancer Research Center, Portland, OR USA; 113 Department of Medical Oncology, Istituto Europeo di Oncologia, Milan, Italy; 114 Department of Pathology, AZ Turnhout, Turnhout, Belgium; 115 Department of Pathology, St Vincent’s University Hospital and University College Dublin, Dublin, Ireland; 116 Department of Computational and Systems Biology, University of Pittsburgh, Pittsburgh, PA USA; 117 Azienda AUSL, Regional Hospital of Aosta, Aosta, Italy; 118 Department of Clinical Medicine, Macquarie University, Sydney, NSW Australia; 119 HistoGeneX NV, Antwerp, Belgium and AZ Sint-Maarten Hospital, Mechelen, Belgium; 120 Oncology Clinical Development, Bristol-Myers Squibb, Princeton, NJ USA; 121 Institut für Pathologie, Hamburg, Germany UK; 122 Department of Medicine, Vanderbilt University Medical Centre, Nashville, TN USA; 123 Department of Cancer Biology, Mayo Clinic, Jacksonville, FL USA; 124 Department of Oncology, Mayo Clinic, Rochester, MN USA; 125 Roche, Tucson, AZ USA; 126 Tumor Pathology Department, Maria Sklodowska-Curie Memorial Cancer Center, Gliwice, Poland; 127 Pathology and Tissue Analytics, Pharma Research and Early Development, Roche Innovation Center Munich, Penzberg, Germany; 128 Sunnybrook Health Sciences Centre, Toronto, ON Canada; 129 Tehran University of Medical Sciences, Tehran, Iran; 130 Oncology Biomarker Development, Genentech-Roche, San Francisco, CA USA; 131 Division of Medical Oncology, Department of Medicine, University of Colorado Anschutz Medical Campus, Aurora, CO USA; 132 Department of Pathology, Hospital de Oncología Maria Curie, Buenos Aires, Argentina; 133 Directorate of Surgical Pathology, SA Pathology, Adelaide, Australia; 134 Department of Pathology, University Hospital Antwerp, Edegem, Belgium; 135 Translational Health Sciences, Department of Cellular Pathology, North Bristol NHS Trust, University of Bristol, Bristol, UK; 136 Medical Oncology, Department of Medicine, Cedars-Sinai Medical Center, Los Angeles, CA USA; 137 Helsinki University Central Hospital, Helsinki, Finland; 138 Department of Clinical Pathology, Akademiska University Hospital, Uppsala, Sweden; 139 International Agency for Research on Cancer (IARC), World Health Organization, Lyon, France; 140 Department of Pathology, Sanatorio Mater Dei, Buenos Aires, Argentina; 141 Institute of Pathology, Medical University of Graz, Graz, Austria; 142 Division of Medical Oncology, National Cancer Centre Singapore, Singapore, Singapore; 143 R&D UNICANCER, Paris, France; 144 Precision Medicine, Oncology R&D, AstraZeneca, Gaithersberg, MD USA; 145 Breast Unit, Champalimaud Clinical Centre, Lisboa, Portugal; 146 Department of Pathology, Massachusetts General Hospital, Boston, MA USA; 147 Department of Laboratory Medicine and Pathology, Mayo Clinic, Rochester, USA; 148 Department of Oncology and Pathology, Karolinska Institutet and University Hospital, Solna, Sweden; 149 Department of Medicine, Clinical Division of Oncology, Comprehensive Cancer Centre Vienna, Medical University of Vienna, Vienna, Austria; 150 Leicester Cancer Research Centre, University of Leicester, Leicester, and MRC Toxicology Unit, University of Cambridge, Cambridge, UK; 151 Merck & Co., Inc, Kenilworth, NJ USA; 152 GHI Le Raincy-Montfermeil, Chelles, Île-de-France, Montfermeil, France; 153 Department of Pathology, Gustave Roussy, Grand Paris, France; 154 Departments of Medicine and Cancer Biology, Vanderbilt University Medical Centre, Nashville, TN USA; 155 Vm Scope, Berlin, Germany; 156 Department of Pathology, Breast Pathology Section, Northwestern University, Chicago, IL USA; 157 Department of Biometrics, The Netherlands Cancer Institute, Amsterdam, The Netherlands; 158 Cancer Biomarkers Working Group, Faculty of Medicine and Pharmacy, Université Mohamed Premier, Oujda, Morocco; 159 Pathology Department, Stanford University Medical Centre, Stanford, CA USA; 160 Center for Pharmacogenomics and Fudan-Zhangjiang, Center for Clinical Genomics School of Life Sciences and Shanghai Cancer Center, Fudan University, Shanghai, China; 161 GROW - School for Oncology and Developmental Biology, Maastricht University Medical Centre and Department of Pathology, Maastricht University Medical Centre, Maastricht, The Netherlands; 162 Biorepository and Tissue Technology Shared Resources, University of California San Diego, San Diego, CA USA; 163 Department of Pathology, Gustave Roussy, Villejuif, France; 164 Departments of Epigenetics and Molecular Carcinogenesis, The University of Texas MD Anderson Cancer Center, Houston, TX USA; 165 Hospital das Clínicas, Sao Paulo, Brasil; Department of Pathology, Faculty of Medicine, University of São Paulo, Sao Paulo, Brasil; 166 Department of Medical Biology and Pathology, Gustave Roussy Cancer Campus, Villejuif, France; 167 Department of Pathology, Academic Medical Center, Amsterdam, The Netherlands; 168 Institut Jules Bordet, Universite Libre de Bruxelles, Brussels, Belgium; 169 Roche Tissue Diagnostics, Digital Pathology, Santa Clara, CA USA; 170 Department of Pathology, Microbiology and Immunology, Vanderbilt University Medical Centre, Nashville, TN USA; 171 Division of Biostatistics, Dana-Farber Cancer Institute, Boston, MA USA; 172 Roche Diagnostics Information Solutions, Belmont, CA USA; 173 Department of Anatomical Pathology, Royal Melbourne Hospital, Parkville, VIC Australia; 174 Vernon Cancer Center, Newton-Wellesley Hospital, Newton, MA USA; 175 Department of Pathology, Cliniques Universitaires Saint-Luc Bruxelles, Brussels, Belgium; 176 Breast Center, Dept. OB&GYN and CCC (LMU), University of Munich, Munich, Germany; 177 Division of Hematology-Oncology, Beth Israel Deaconess Medical Center, Boston, MA USA; 178 University of Leuven, Leuven, Belgium; 179 Department of Pathology, Institut Jules Bordet, Université Libre de Bruxelles, Brussels, Belgium; 180 Department of Surgical Pathology and Biopathology, Jean Perrin Comprehensive Cancer Centre, Clermont-Ferrand, France; 181 Johanniter GmbH - Evangelisches Krankenhaus Bethesda Mönchengladbach, West German Study Group, Mönchengladbach, Germany; 182 Molecular Oncology Group, Vall d’Hebron Institute of Oncology, Barcelona, Spain; 183 Department of Pathology, University of Marburg, Marburg, Germany; 184 Department of Histopathology, Manipal Hospitals Dwarka, New Delhi, India; 185 Department of Anatomical Pathology, St Vincent’s Hospital Melbourne, Fitzroy, VIC Australia; 186 Cancer Immunotherapy Trials Network, Central Laboratory and Program in Immunology, Fred Hutchinson Cancer Research Center, Seattle, WA USA; 187 Clinical Trial Service Unit & Epidemiological Studies Unit, University of Oxford, Oxford, UK; 188 Department of Radiation Oncology, Odette Cancer Centre, Sunnybrook Research Institute, Toronto, ON Canada; 189 The Cancer Research Program, Garvan Institute of Medical Research, Darlinghurst, Australian Clinical Labs, Darlinghurst, NSW Australia; 190 Georgetown University Medical Center, Washington, DC USA; 191 Department of Molecular and Experimental Medicine, Avera Cancer Institute, Sioux Falls, SD USA; 192 Translational Medicine, Bristol-Myers Squibb, Princeton, NJ USA; 193 Department of Pathology, Ahfad University for Women, School of Medicine, Omdurman, Sudan; 194 Guy’s Hospital, London, UK; King’s College London, London, UK; 195 Department of Breast Surgery, Sichuan Cancer Hospital & Institute, Sichuan Cancer Center, School of Medicine, University of Electronic Science and Technology of China, Chengdu, China; 196 Department of Health Sciences, University of Florence, Florence, Italy; 197 Department of Oncology, Champalimaud Clinical Centre, Lisbon, Portugal; 198 Department of Pathology, Fundación Valle del Lili, Cali, Colombia; 199 Department of Development and Regeneration, Laboratory of Experimental Urology, KU Leuven, Leuven, Belgium; 200 Department of Medical Oncology, Austin Health, Heidelberg, VIC Australia; 201 Department of Surgery, Kansai Medical School, Hirakata, Japan; 202 Pathology Department, H.U. Vall d’Hebron, Barcelona, Spain; 203 Division of Bioinformatics and Biostatistics, U.S. Food and Drug Administration, Silver Spring, MD USA; 204 Department of Pathology and Laboratory Medicine, Rhode Island Hospital and Lifespan Medical Center, Providence, RI USA; 205 Université Paris-Est, Créteil, France; 206 Praava Health, Dhaka, Bangladesh; 207 Department of Pathology, Medical University of Vienna, Vienna, Austria

**Keywords:** Immunosurveillance, Prognostic markers

## Abstract

Stromal tumor-infiltrating lymphocytes (sTILs) are important prognostic and predictive biomarkers in triple-negative (TNBC) and HER2-positive breast cancer. Incorporating sTILs into clinical practice necessitates reproducible assessment. Previously developed standardized scoring guidelines have been widely embraced by the clinical and research communities. We evaluated sources of variability in sTIL assessment by pathologists in three previous sTIL ring studies. We identify common challenges and evaluate impact of discrepancies on outcome estimates in early TNBC using a newly-developed prognostic tool. Discordant sTIL assessment is driven by heterogeneity in lymphocyte distribution. Additional factors include: technical slide-related issues; scoring outside the tumor boundary; tumors with minimal assessable stroma; including lymphocytes associated with other structures; and including other inflammatory cells. Small variations in sTIL assessment modestly alter risk estimation in early TNBC but have the potential to affect treatment selection if cutpoints are employed. Scoring and averaging multiple areas, as well as use of reference images, improve consistency of sTIL evaluation. Moreover, to assist in avoiding the pitfalls identified in this analysis, we developed an educational resource available at www.tilsinbreastcancer.org/pitfalls.

## Introduction

Despite the complexity of the immune system and intricate interplay between tumor and host antitumor immunity, detection of stromal tumor-infiltrating lymphocytes (sTILs), as quantified by visual assessment on routine hematoxylin and eosin (H&E)-stained slides, has emerged as a robust prognostic and predictive biomarker in triple-negative and HER2-positive breast cancer^1–3^. Stromal TILs are defined as mononuclear host immune cells (predominantly lymphocytes) present within the boundary of a tumor that are located within the stroma between carcinoma cells without directly contacting or infiltrating tumor cell nests. Stromal TILs are reported as a percentage, which refers to the percentage of stromal area occupied by mononuclear inflammatory cells over the total stromal area within the tumor (i.e., not the percentage of cells in the stroma that are lymphocytes). Intratumoral TILs (iTILs), on the other hand, are defined as lymphocytes within nests of carcinoma having cell-to-cell contact with no intervening stroma. Initial studies of TILs in breast cancer evaluated stromal and intratumoral lymphocytes separately and while both correlated with outcome, sTILs were more prevalent, more variable in amount and shown to be more reproducibly assessed^4–7^. As such, recommendations for standardized assessment of TILs in breast cancer by the International Immuno-Oncology Biomarker Working Group (also referred to as TIL-Working Group, or TIL-WG in the manuscript; www.tilsinbreastcancer.org) recommend assessing sTILs whilst strictly adhering to the definition as outlined above^8^.

Stromal TILs are prognostic for disease-free and overall survival in early triple-negative breast cancers treated with standard anthracycline-based adjuvant chemotherapy^4–6,9,10^. High levels of sTILs are associated with improved outcome and increased response to neoadjuvant therapy in both triple-negative and HER2-positive breast cancers^7,11–14^. Recently, experts at the 16th St. Gallen International Breast Cancer Conference endorsed routine reporting of sTILs in triple-negative breast cancer^15^. Studies involving or evaluating prognosis should now include the evaluation of sTILs.

The expanding role sTILs play in breast cancer research, prognosis and increasingly patient management, is predicated on accurate assessment of sTILs. The pivotal studies cementing the prognostic and predictive role of sTILs have been performed by visual assessment on H&E-stained slides according to published recommendations^8^. In the future, advances in machine learning may open the door to automated sTIL assessment^16^. Until that point, however, the onus for accurate sTIL assessment falls upon the pathologist.

Management of breast cancer is continually evolving. In contrast to the excisional biopsies of previous decades, an initial diagnosis of breast cancer is now routinely rendered on needle biopsy specimens. These small biopsies are particularly susceptible to influence of tumor heterogeneity, limited tumor sampling and technical artifacts such as crushing. Studies assessing concordance of TILs between core needle biopsies and matched surgical specimens (lumpectomy or mastectomy) report higher average TIL counts (4.4–8.6% higher) in the surgical specimens^17,18^. The difference in TIL scores between biopsies and surgical specimens was found to be reduced when the number of cores was increased^18^, suggesting tumor heterogeneity as a contributing factor. Not specifically addressed was the tissue reaction and inflammatory infiltrate associated with the biopsy procedure itself. No increase in TIL scores within the surgical specimens was seen when surgery was performed within 4 days of the biopsy procedure. Conversely, surgery performed more than 4 days post biopsy was an independent factor correlating with higher TILs in the surgical specimen^17^. This corresponds to the timing of chronic inflammatory infiltrates in wound healing. It should be noted, however, that in most contemporary practice settings the delay between biopsy and surgery is several weeks and per the recommended guidelines, areas of scarring should be excluded from sTIL assessment. The inflammation associated with wound healing is physically limited closely to the healing area and does not spread extensively into the tumor itself or surrounding stroma. Thus the impact of the biopsy procedure on sTIL levels in the surgical specimen is likely minimal.

Routine use of neoadjuvant therapy is increasingly common in triple-negative and HER2-positive breast cancers. These trends necessitate that sTIL assessment be performed on small biopsy samples and, in the absence of complete pathological response, on postneoadjuvant excision specimens without compromising accuracy. High levels of sTILs in residual tumor post neoadjuvant therapy is associated with improved outcome in TNBC^19,20^. As neoadjuvant samples possess distinct challenges, separate recommendations for assessing TILs in residual disease after neoadjuvant therapy have been published^21^.

Breast cancers show wide variation in morphology, particularly in tumor cellularity and amount of tumor stroma. Two tumors of the same size may exhibit the same absolute numbers of stromal lymphocytes but have a different percentage of sTILs due to the stromal content as a proportion of tumor area. High-grade tumors can show extensive central necrosis with only a thin rim of viable tumor resulting in minimal assessable tumor stroma even in large resection specimens. Other inflammatory cells are not infrequently seen infiltrating tumor stroma, including neutrophils, eosinophils and macrophages, resulting in a more cellular appearance and rendering assessment of stromal TIL density more challenging. Apoptotic cells can mimic lymphocytes. Poor fixation and technical artifacts in cutting and staining are recognized to compromise sTIL assessment. Ill-defined tumor borders and widely separated nests of tumor result in variability in defining what constitutes tumor stroma. Preexisting lymphocytic aggregates surrounding normal ducts and lobules, vessels or ductal carcinoma in situ (DCIS) can also confound assessment. Heterogeneity in sTIL distribution both within the tumor and at the invasive front versus the central tumor all contribute to variation in pathologist sTIL assessment.

In an effort to identify the sources of variation in assessment of sTILs, we analyzed data and images from three-ring studies performed by TIL-WG pathologists specifically evaluating concordance in sTIL evaluation in breast cancer^22,23^. Based on the findings of this analysis we designed an educational resource available via the International Immuno-Oncology Working Group website at www.tilsinbreastcancer.org/pitfalls to assist pathologists in avoiding the different types of pitfalls identified. In addition, we evaluated the impact of sTIL discrepancy on outcome estimation using the data of a pooled analysis of 9 phase III clinical trials^9^.

## Results

### Identification of cases demonstrating variability using ring studies by the TIL-Working Group

**Fig. 1:**
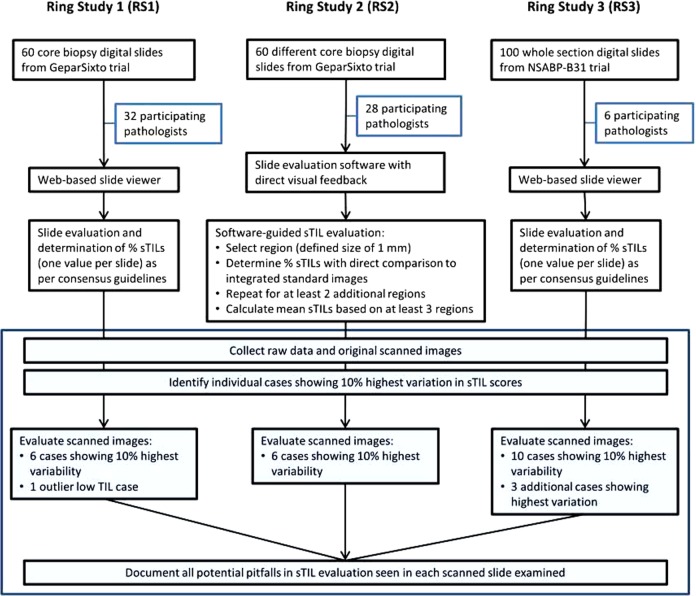
Study flow diagram. Raw data and original scanned images from 3 previously performed ring studies were evaluated (shaded Box 1).

**Fig. 2:**
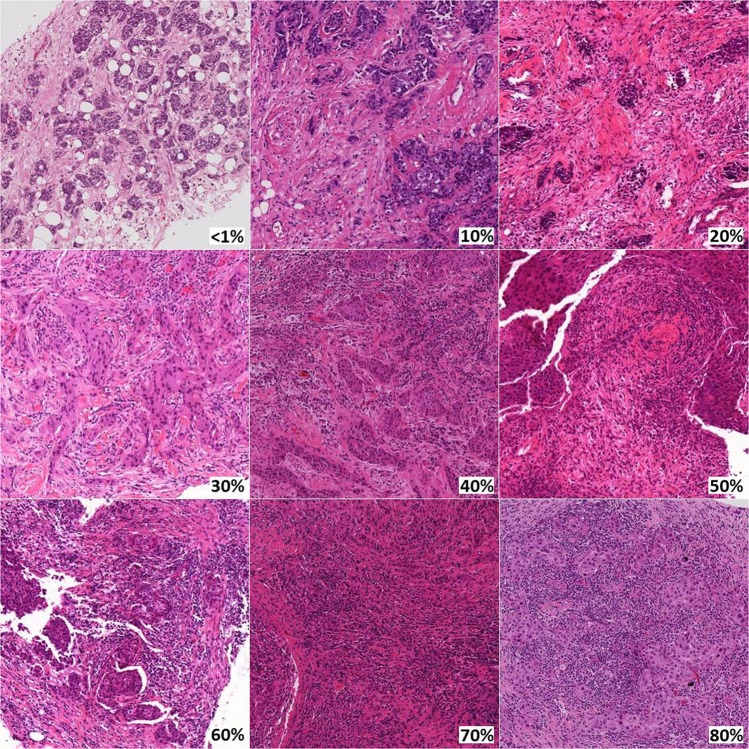
Reference images representing percent sTIL scores. Available at www.tilsinbreastcancer.org.

Three-ring studies evaluating concordance of sTIL assessment in breast cancer were analyzed (Fig. 1). In the first ring study, 32 pathologists evaluated 60 scanned breast cancer core biopsy slides^22^. This international group of pathologists from 11 different countries were all members of the TIL Working Group. Some had a special interest or subspecialty training in breast pathology, while others were general surgical pathologists, illustrating the wide applicability of the approach. The only instructions given to the scoring pathologists were to read and use the TIL assessment guidelines published by the TIL working group^8^. The second ring study was an extension of the first study using a more formalized approach. A subset of 28 of the original 32 pathologists participated and scored 60 different scanned breast cancer core biopsy slides. In this study, each pathologist identified and scored at least three separate 1 mm^2^ regions on each slide, representing the range of sTIL variability and averaged the results into a final score. Additionally, reference images representing different sTIL percentages were integrated into the evaluation process (Fig. 2)^22^. The last ring study was performed by six TIL-WG pathologists who independently scored 100 scanned whole section (excision specimen) breast cancer cases^23^.

**Fig. 3:**
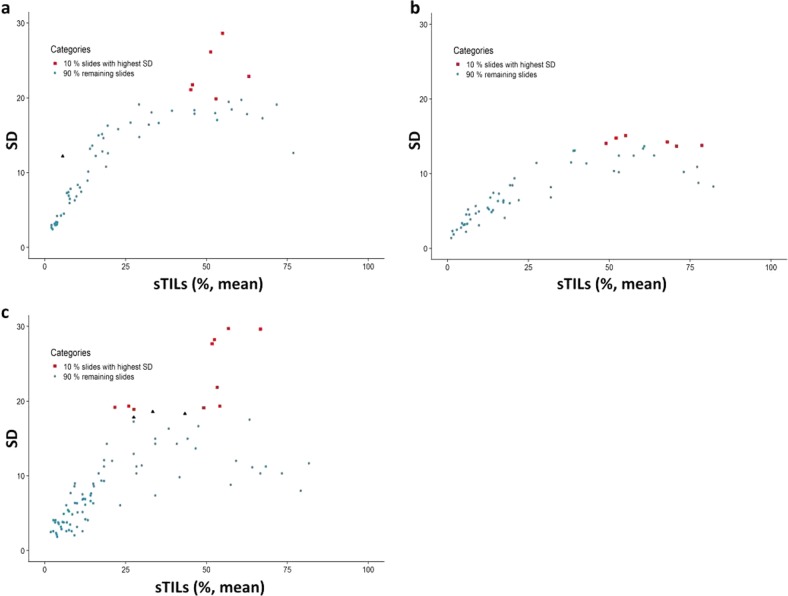
Standard deviation as a function of mean across all sTILs scores for each slide in 3 ring studies assessing concordance amongst pathologists. **a** Ring study 1, 32 pathologists evaluated 60 scanned core biopsy specimens. **b** Ring study 2, 28 pathologists evaluated 60 scanned core biopsy specimens. **c** Ring study 3, 6 pathologists evaluated 100 scanned whole section specimens. 10% of cases in each study showing the greatest variability in sTIL scores are shown as red squares. Black triangles identify additional cases identified for slide assessment.

In total, results from 220 slides were included for statistical analysis (60 each from ring studies 1 and 2, and 100 from ring study 3). The standard deviation for sTIL scores for each slide is shown in Fig. 3. When comparing across studies, ring study 2 shows the least variation in sTIL scores between pathologists. The cases with the 10% greatest standard deviation were identified (Fig. 3 red squares) and the original scanned slides of the cases were reviewed to identify factors contributing to discordant sTIL assessment in these cases. Additionally, in Ring Study 1, a single outlier case in the low sTIL range was also evaluated (Fig. 3a black triangle). From Ring Study 3, three additional cases showing large standard deviation were also included in the scanned slide assessment (Fig. 3c black triangles). Overall, a total of 26 original scanned images were reviewed by ZK (ring studies 1 and 2) and RK (ring study 3) from cases identified as particularly problematic (i.e., showing high variability) in sTIL assessment.

Box 1 Key Points

- Stromal TILs are mononuclear cells (predominantly lymphocytes) present within the boundary of a tumor that are located within the stroma between carcinoma cells without directly contacting the carcinoma cell nests.
- Heterogeneity in sTIL distribution is the main contributing factor to variability in assessment.
- Two key factors improve consistency of sTIL results:
  - ∘ Scoring multiple areas in heterogeneous tumors and averaging results.
  - ∘ Use of reference images.
- Poor sample processing or fixation can increase histological artifacts and compromise assessment of sTILs.
- Careful adherence to the definition and morphology of sTILs is required to avoid scoring stromal areas outside of the tumor boundary and mistaken classification of artifacts, mitotic bodies, etc as sTILs.

### Analysis of scoring variance between pathologists

Table 1 shows the intraclass correlation coefficient (ICC) and concordance rate among pathologists for each of the 3 studies. The ICC is the proportion of total variance (in measurements across patients and laboratories) that is attributable to the biological variability among patients’ tumors, while 1 – ICC is the proportion attributable to pathologist variability. The ICC has a range from 0 to 1 with a score of 1 having the maximum agreement. Concordance rates were evaluated comparing different sTIL cutpoints: <1 vs ≥1%; <5 vs ≥5%; <10 vs ≥10%; <30 vs ≥30%; <75 vs ≥75% for each pathologist by comparing all pairs of pathologists.

**Table 1:** Comparison of intraclass correlation coefficient and pair-wise observer concordance rate for 3 ring studies.

	Ring study 1	Ring study 2	Ring study 3
***ICC***	0.7 (0.62–0.78)	0.89 (0.85–0.92)	0.76 (0.69–0.83)
*Concordance rates*^a^			
TILs <1 vs ≥1%	0.94 (±0.08)	0.94 (±0.04)	0.91 (±0.06)
TILs <5 vs ≥5%	0.83 (±0.09)	0.89 (±0.05)	0.84 (±0.1)
TILs <10 vs ≥10%	0.77 (±0.08)	0.86 (±0.05)	0.79 (±0.06)
TILs <30 vs ≥30%	0.81 (±0.08)	0.93 (±0.03)	0.87 (±0.04)
TILs <75 vs ≥75%	0.90 (±0.06)	0.92 (±0.03)	0.94 (±0.03)

ICC intraclass correlation coefficient, TILs tumor-infiltrating lymphocytes.

^a^The concordance of all pairs of pathologists was calculated for five different TIL-groups. The values in the table are the sample mean and sample standard deviation of these concordance rates for all pairs of pathologists in each study.

The ICC was highest in ring study 2 compared to the other studies. Ring study 2 specifically sought to mitigate effects of sTIL heterogeneity with assessment of 3 separate areas and intra-pathologist scoring bias by necessitating use of standardized percentage sTIL reference images.

### Evaluation of sources of variability in the three-ring studies

The scanned images of the H&E-stained slides from the most discordant cases in each of the 3 ring studies were evaluated to identify the histological factors contributing to the variation in sTIL assessment. In total 26 original scanned images were reviewed—7 from ring study 1, 6 from ring study 2 and 13 from ring study 3. Often multiple factors were present in each slide.

### Heterogeneity in sTIL distribution

**Fig. 4:**
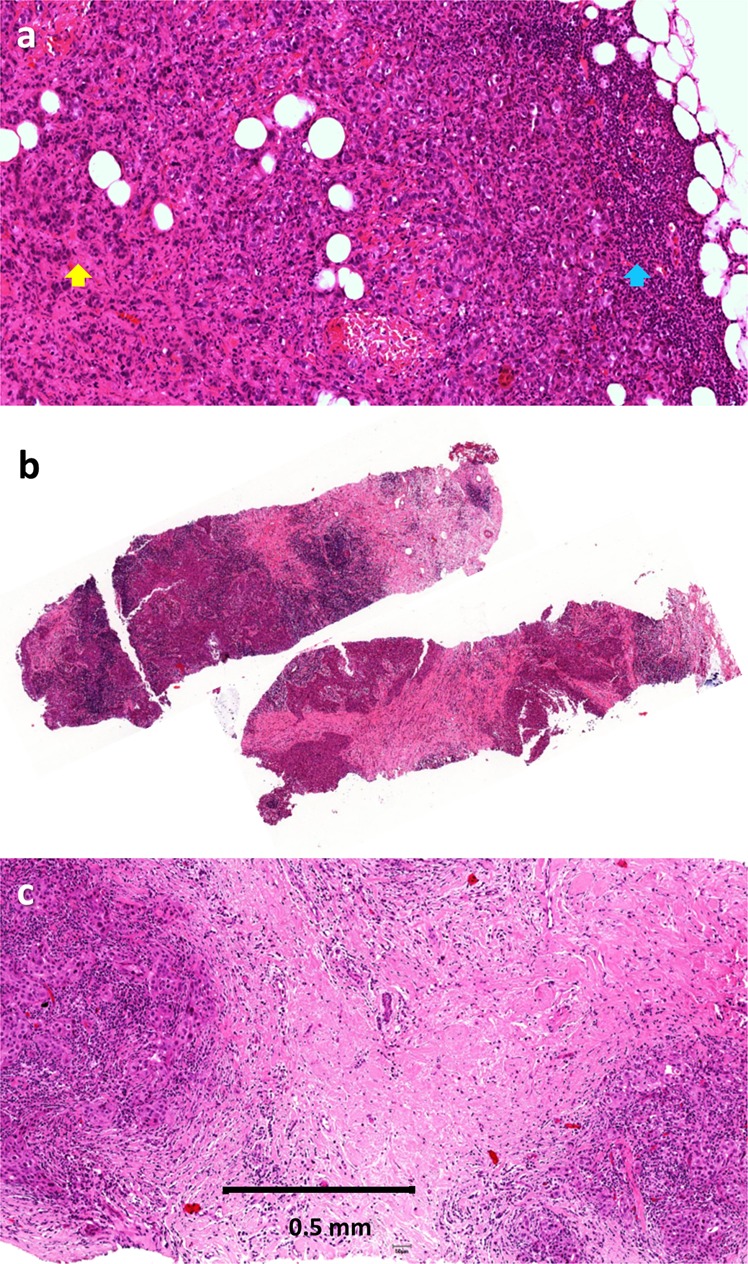
Heterogeneity in sTIL distribution as a cause of variation in sTIL assessment in breast cancer. Different examples of heterogeneity include **a** increased sTILs at the leading edge (blue arrow) compared to the central tumor (yellow arrow); **b** marked heterogeneity in sTIL density within the tumor; and **c** variably spaced apart clusters of cancer cells with a dense tight lymphocytic infiltrate separated by collagenous stroma with sparse infiltrate.

Heterogeneity in sTIL distribution was identified as a major contributing factor in all of the ring studies and as the most prevalent challenge in ring studies 1 and 2 (Table 2; Fig. 4). Based on review of the most variable cases, increased sTIL density at the leading edge versus central tumor were contributing factors in 43%, 17% and 54% of cases in ring studies 1 through 3, respectively (Fig. 4a); and marked heterogeneity of sTIL density within the tumor was identified in 29% cases in ring study 1 only (Fig. 4b). Whereas in ring studies 1 and 3 pathologists provided a global sTIL assessment based simply on the published scoring recommendations^8^, ring study 2 specifically addressed the issue of sTIL heterogeneity by requiring separate scoring of at least 3 distinct areas of the tumor representing the range of sTIL density. Additionally, matching the tumor area observed with reference percent sTIL images were a necessary part of the evaluation. Our analysis supports that scoring and averaging multiple areas aids in providing a more consistent result between pathologists. One issue not resolved by this technique is the scenario of a tumor comprised of variably spaced apart clusters of epithelial cells with a dense lymphocytic aggregate associated with each cluster of epithelial nests but sparse infiltrate between the clusters (Fig. 4c). This pattern was identified as a contributing factor in 29% of highly discordant cases in ring study 1, 50% of discordant cases in ring study 2 and no cases in ring study 3. There appears to be uncertainty amongst pathologists in this situation as to whether to only include the stroma associated with—but not touching—tumor epithelium (showing high sTIL density) or all stroma within the tumor mass including stroma intervening between spaced apart clusters of malignant epithelium (showing low sTIL density). This uncertainty increases variability in sTIL assessment and would be reduced by strict adherence to the definition of sTILs provided in the introduction. All stroma within a single tumor is to be included within the sTIL assessment. In this situation, both the higher density areas in close proximity to tumor cells and the lower density areas located between epithelial clusters should be included. One notable exception is a tumor with a central hyalinized scar, where the acellular scar tissue should be excluded from sTIL assessment.

**Table 2:** Pitfalls in sTIL assessment in breast cancer slides identified from cases showing the highest variation in 3 ring studies (RS)—heterogeneity of lymphocyte distribution.

Pitfall	Frequency seen	Recommendation
**Heterogeneity**	**15/26 (58%)**	
Increased sTILs at the leading edge compared to central tumor (Fig. 4a)	RS1: 3/7 (43%) RS2: 1/6 (17%) RS3: 7/13 (54%)	Increased density of lymphocytes at the leading front should be included as long as the lymphocytes are within the boundary of the tumor. Scoring multiple areas and averaging the results can help with heterogeneous tumors.
Marked hterogeneity in sTIL density within the tumor (Fig. 4b)	RS1: 2/7 (29%) RS2: 0 RS3: 0	All stroma within the boundary of a single tumor is included in sTIL assessment. Scoring multiple distinct areas encompassing the range of sTIL density and averaging the results can assist in providing a more reproducible overall sTIL score.
Variably spaced apart clusters of cancer cells with a dense tight lymphocytic infiltrate separated by collagenous stroma with sparse infiltrate (Fig. 4c)	RS1: 2/7 (29%) RS2: 3/6 (50%) RS3: 0	All stroma within a single tumor is included within the sTIL assessment. In this situation, both the higher density areas closely associated with (but not touching) epithelial clusters and the lower density areas located between epithelial clusters are included. [The exception is a central hyalinized scar, which is excluded from scoring.] Scoring multiple areas and averaging the results can help with heterogeneous tumors.

*RS1* Ring Study 1, *RS2* Ring Study 2, *RS3* Ring Study 3.

### Technical factors

**Fig. 5:**
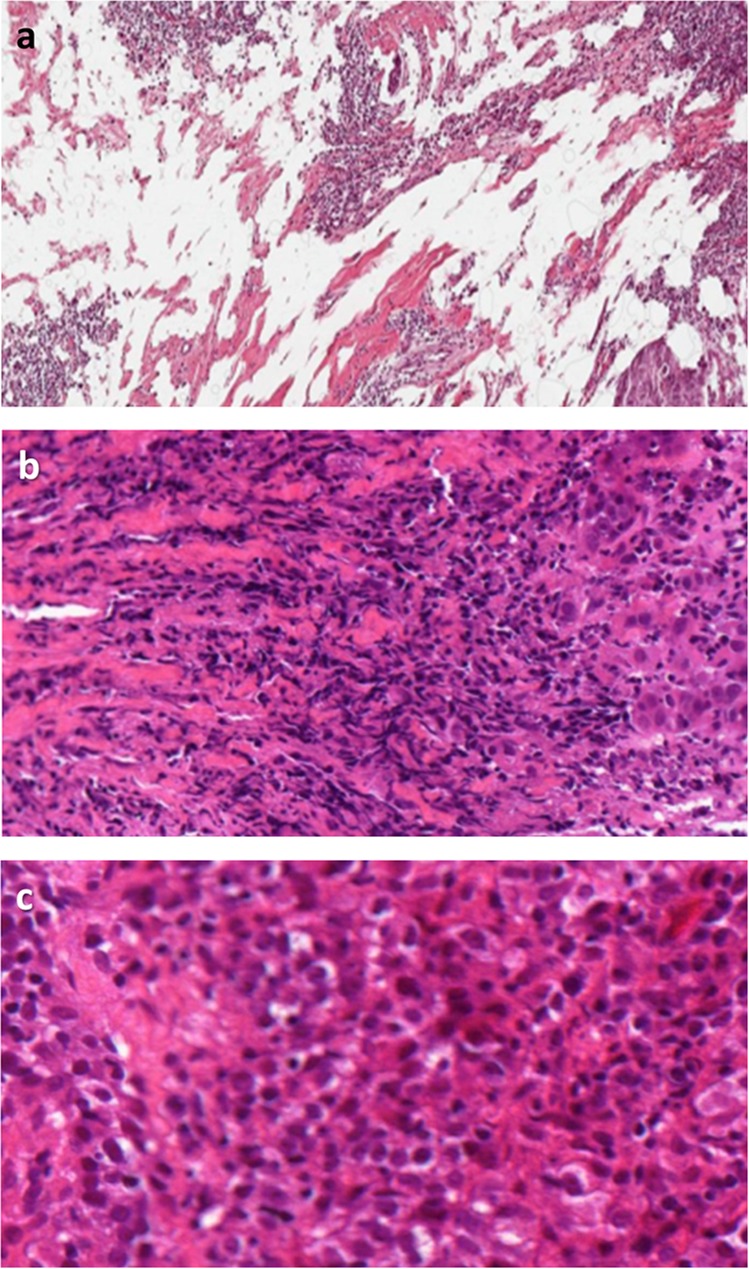
Technical factors as a cause of variation in sTIL assessment in breast cancer. Examples of different technical factors include **a** a poor quality slide as can be seen secondary to prolonged ischemic time, poor fixation or issues during processing; **b** crush artifact; and **c** out-of-focus scan.

Technical factors were the next largest source of discordance (Table 3; Fig. 5). Poor quality slides with histological artifacts, as can be seen secondary to prolonged ischemic time, poor fixation, issues during processing, embedding or microtomy were identified as a contributing factor for discordance in 85% of the most discordant scanned slides from ring study 3 (Fig. 5a). In contrast, this was not deemed a contributing factor in any of the cases from ring studies 1 or 2. These results are highly skewed based on the studies assessed. Ring study 3 used a subset of H&E slides from NSABP-B31, an older completed trial evaluating benefit of trastuzumab in early HER2-positive breast cancer, which started accrual in February 2000 across multiple centers. These were excision specimens undergoing local community tissue processing. Variable ischemic and fixation times subsequently affected the integrity of stromal connective tissue which is critical in sTIL assessment. Ring studies 1 and 2 used pretherapeutic core biopsies from the neoadjuvant GeparSixto trial, which accrued between August 2011 and December 2012. Fixation and ischemic time are less likely to have been an issue in these samples, which (i) as biopsy samples are immediately placed in formalin without requirement for serial sectioning and can be processed in a timely fashion and (ii) were procured at a time when the preanalytic variables had become substantially better understood and new recommendations widely adopted. Not to mention, H&E stains fade with passage of time, which itself impacts the ability to produce quality scanned images. In the current era, with awareness and adoption of standardization and monitoring of preanalytical and analytical variables, poor quality H&E slides should no longer be acceptable. Nonetheless, challenges remain and variations in practice can result in poorly processed specimens that are likely to directly and negatively impact sTIL assessment. Crush artifact, which is more commonly seen in core biopsy samples, was seen in 1 case overall in ring study 1 (14%) (Fig. 5b).

**Table 3:** Pitfalls in sTIL assessment in breast cancer slides identified from cases showing the highest variation in 3 ring studies (RS)—technical factors.

Pitfall	Frequency seen	Recommendation
**Technical factors**	**13/26 (50%)**	
Poor quality slides / Histological artifacts secondary to prolonged ischemic time, poor fixation or issues during processing (Fig. 5a)	RS1: 0 RS2: 0 RS3: 11/13 (85%)	Thankfully, in the current era, with greater awareness and monitoring of preanalytical and analytic variables, these sorts of poor quality H&E slides should not be an issue. If presented with such a case, only intact, morphologically assessable areas should be included in sTIL score. If applicable, one can cut and stain an additional section or select a different block for assessment.
Crush artifact (Fig. 5b)	RS1: 1/7 (14%) RS2: 0 RS3: 0	More commonly seen in biopsy samples, crush artifact can compromise sTIL assessment. Areas of crushing should be excluded from sTIL evaluation.
Out-of-focus scan (Fig. 5c)	RS1: 1/7 (14%) RS2: 1/6 (17%) RS3: 0	As part of a study one may struggle with scoring an out-of-focus scan. In clinical practice, however, particularly as sTILs are poised to impact patient management, there is no good justification to not rescan the slide. If this is not a possibility most computer programs have some capability of image correction.

*RS1* Ring Study 1, *RS2* Ring Study 2, *RS3* Ring Study 3.

Out-of-focus scans were identified in 1 case each in ring study 1 (14%) and ring study 2 (17%) (Fig. 5c). In clinical practice, particularly as sTILs are poised to impact patient management, an out-of-focus slide should be rescanned before scoring. Notably, this highlights an obstacle to incorporation of whole slide imaging in routine practice. Consistent focus quality remains an issue requiring dedicated support staff for loading, scanning, reviewing and rescanning if necessary^24^.

### Including wrong area or cells

**Fig. 6:**
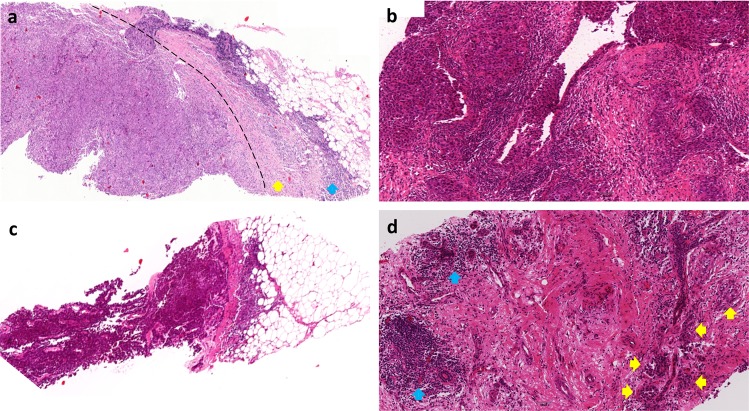
Scoring the wrong area as a cause of variation in sTIL assessment in breast cancer. Scenarios where there may be challenges in deciding which areas to score include **a** difficulty defining the tumor boundary (dashed line) and including fibrous scars (yellow arrow) or lymphoid aggregates (blue arrow) beyond the invasive front; **b** including lymphocytes surrounding ductal carcinoma in situ (DCIS) which may be difficult to distinguish from invasive carcinoma; **c** including lymphocytes associated with an encapsulated papillary carcinoma component of a tumor; and **d** including lymphocytes surrounding benign glands. Shown is invasive carcinoma (yellow arrows) surrounding a benign lobule with associated lymphocytes; adjacent benign lobules (blue arrows) show dense lymphoid aggregates identify the lymphocytic infiltrate to be related to the entrapped lobule rather than the carcinoma.

**Fig. 7:**
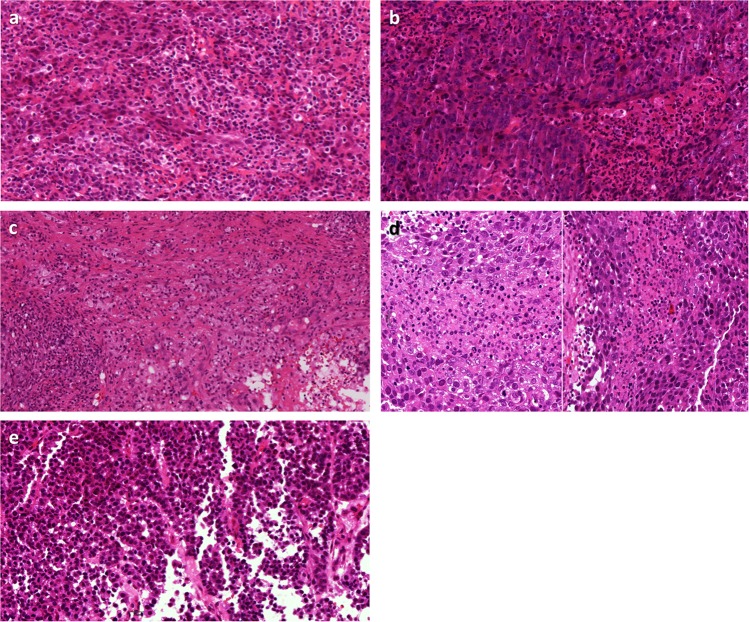
Scoring the wrong cells as a cause of variation in sTIL assessment in breast cancer. Examples where the wrong cells are scored include **a** counting intratumoral TILs (iTILS); **b** counting neutrophils; **c** counting histiocytes; **d** misinterpreting apoptotic cells as lymphocytes; and **e** artifactual falling apart of cells mimicking TILs.

Variability in defining the tumor boundary and scoring stroma outside of the tumor boundary appears to have been a contributing factor for variation in 33% of highly discordant cases in ring study 2 and 15% of cases in ring study 3 (Table 4; Fig. 6a). The discordant cases also highlighted situations of including lymphocytes associated with DCIS (2 cases ring study (RS)1, 1 case RS2) (Fig. 6a), lymphocytes associated with a component of the tumor showing features of an encapsulated papillary carcinoma (1 case RS1) (Fig. 6b), and lymphocytes associated with benign terminal duct lobular units (1 case RS1) (Fig. 6d). Difficulty distinguishing iTILs from sTILs factored into 2 cases (29%) in ring study 1 and 1 case (17%) in ring study 2 (Fig. 7a). Also identified in ring study 1 was 1 case (14%) with prominent stromal neutrophils (Fig. 7b) and 1 case (14%) with stromal histiocytes (Fig. 7c). It is important to assess slides at a sufficiently high power to be able to differentiate between types of immune cells. Neutrophils, eosinophils, basophils, and histiocytes/macrophages are all excluded from sTIL assessment. Two independent cases in ring study 1 demonstrated misinterpretation of apoptotic cells for lymphocytes (Fig. 7d) and artefactual falling apart of tumor cell nests along the edge of a core biopsy mimicking the discohesive appearance of TILs (Fig. 7e). Both are previously noted examples of histomorphologic challenges.

**Table 4:** Pitfalls in sTIL assessment in breast cancer slides identified from cases showing the highest variation in 3 ring studies (RS)—scoring wrong area or cells.

Pitfall	Frequency seen	Recommendation
**Scoring wrong area or cells**	**12/26 (46%)**	
Defining tumor boundary and scoring outside of tumor (Fig. 6a)	RS1: 0 RS2: 2/6 (33%) RS3: 2/13 (15%)	Do not include fibrous scars (image; yellow arrow) or lymphoid aggregates (blue arrow) beyond the invasive front of the tumor.
Including lymphocytes surrounding DCIS (Fig. 6b)	RS1: 2/7 (29%) RS2: 1/6 (17%) RS3: 0	Lymphocytes surrounding DCIS are excluded from assessment of sTILs. Myoepithelial stains can be used if there is doubt as to whether a particular focus is invasive or in situ.
Including lymphocytes associated with encapsulated papillary carcinoma (Fig. 6c)	RS1: 1/7 (14%) RS2: 0 RS3: 0	Only score sTILs associated with conventional invasive carcinoma. Similar to DCIS, lymphocytes associated with encapsulated papillary carcinoma should not be included in the sTIL assessment of the invasive component.
Including lymphocytes surrounding benign glands (Fig. 6d)	RS1: 1/7 (14%) RS2: 0 RS3: 0	Lymphocytes associated with benign lobules or ducts should be excluded from sTIL counts when carcinoma surrounds benign structures. Similar lymphocytic infiltrates outside of the tumor boundary can identify these as not tumor-related.
Including intratumoral TILs (iTILS) (Fig. 7a)	RS1: 2/7 (29%) RS2: 1/6 (17%) RS3: 0	Certain cases show dense lymphocytic infiltrates within the tumor epithelial nests, sometimes obscuring the boundary between tumor cells and stroma. It is important to be aware that intratumoral TILs are excluded from the assessment, which only includes TILs within the intervening stroma. If necessary, a cytokeratin stain may assist with defining tumor from stroma.
Including neutrophils (Fig. 7b)	RS1: 1/7 (14%) RS2: 0 RS3: 0	Only lymphocytes and plasma cells are included in sTIL evaluation. Pathologists should assess slides at a sufficiently high power to be able to differentiate between types of immune cells. Neutrophils, eosinophils, basophils, and histiocytes/ macrophages are all excluded from sTIL assessment.
Including histiocytes (Fig. 7c)	RS1: 1/7 (14%) RS2: 0 RS3: 0	Only lymphocytes and plasma cells are included in sTIL evaluation. Pathologists should assess slides at a sufficiently high power to be able to differentiate between types of immune cells. Neutrophils, eosinophils, basophils, and histiocytes/ macrophages are all excluded from sTIL counts.
Misinterpreting apoptotic cells as lymphocytes (Fig. 7d)	RS1: 1/7 (14%) RS2: 0 RS3: 0	At low power apoptotic cells can mimic lymphocytes. Pathologists should assess slides at a sufficiently high power to differentiate this mimic.
Artifactual falling apart of cells mimicking TILs Fig. 7e)	RS1: 1/7 (14%) RS2: 0 RS3: 0	Artifactual falling apart of tumor cells is more common in biopsy specimens, particularly along the edge. At low power discohesive tumor cells can mimic lymphocytes. Pathologists should assess slides at a sufficiently high power to differentiate this mimic.

*RS1* Ring Study 1, *RS2* Ring Study 2, *RS3* Ring Study 3.

### Limited stroma within tumor for evaluation

**Fig. 8:**
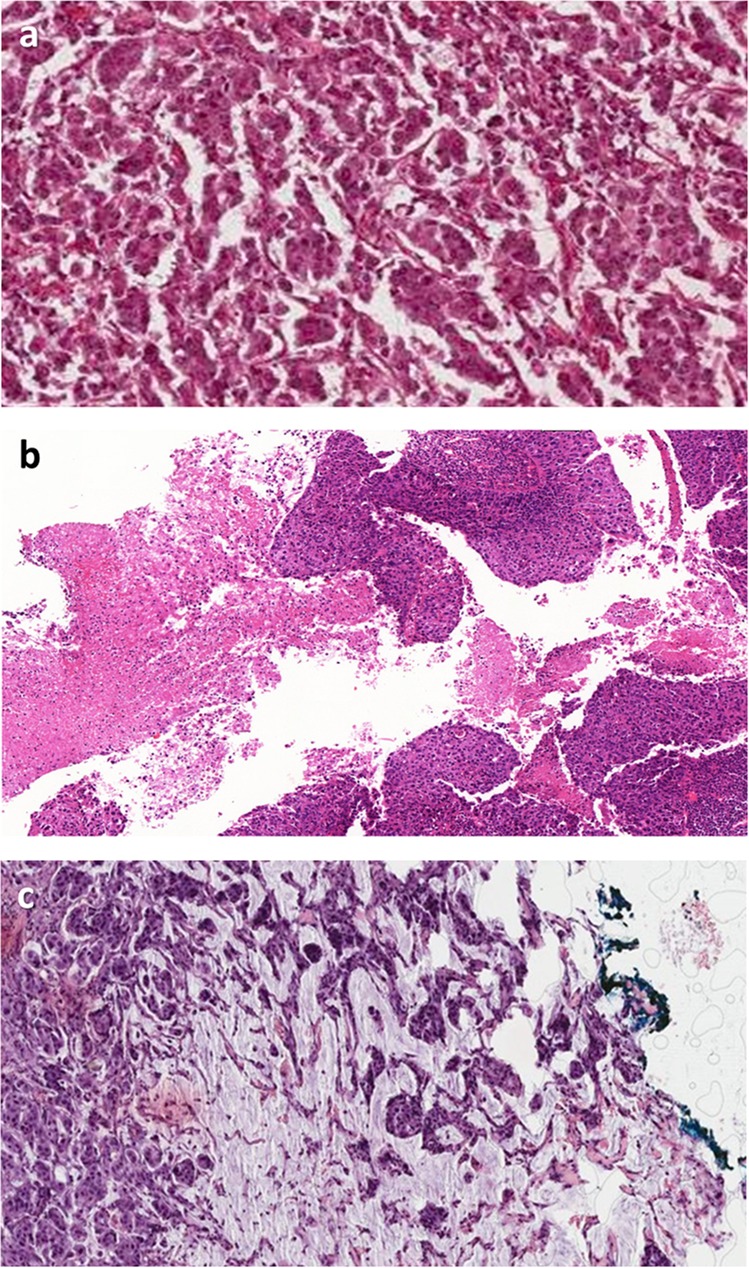
Limited stroma within tumors as a cause of variation in sTIL assessment in breast cancer. Difficulties in sTIL assessment related to stroma include **a** tumor with small volume of intratumoral stroma present for evaluation; **b** large areas of necrosis which decrease scorable stromal component; and **c** mucinous tumors.

An added factor identified was the presence of minimal stroma in the tumor for assessment (Table 5; Fig. 8a). This was identified as a contributing factor in 46% of cases in ring study 3. In a variation, 1 case (14%) in ring study 1 showed extensive tumor necrosis with decreased available stroma for assessment (Fig. 8b). Two cases (15%) of mucinous tumors, each with minimal stroma to assess were identified in ring study 3 (Fig. 8c).

**Table 5:** Pitfalls in sTIL assessment in breast cancer slides identified from cases showing the highest variation in 3 ring studies (RS)—limited tumor stroma.

Pitfall	Frequency seen	Recommendation
**Limited stroma within tumor for evaluation**	**8/26 (31%)**	
Small volume of intratumoral stroma present for evaluation (Fig. 8a)	RS1: 0 RS2: 0 RS3: 6/13 (46%)	Assessing % sTILs is difficult when the denominator is very small. Evaluation should be restricted to areas where there is clear stroma. The leading edge ought to provide at least some tumor stroma for assessment.
Large areas of necrosis (decreases scorable stromal component) (Fig. 8b)	RS1: 1/7 (14%) RS2: 0 RS3: 0	Necrosis and associated granulocytes are excluded from sTIL assessment. Some tumors show extensive necrosis with only a thin rim of viable cells at the periphery. Only lymphocytes associated with viable tumor should be included. Even in highly necrotic tumor, there are typically at least some viable areas along the invasive front.
Mucinous tumors (Fig. 8c)	RS1: 0 RS2: 0 RS3: 2/13 (15%)	Lymphocytes generally are absent within extracellular mucin. Thin septa and fibrous bands are often present providing a stromal component for assessment. Stroma associated with any ‘no special type’ component should be included.

*RS1* Ring Study 1, *RS2* Ring Study 2, *RS3* Ring Study 3.

### Clinical significance of variability in sTIL assessment by pathologists

**Fig. 9:**
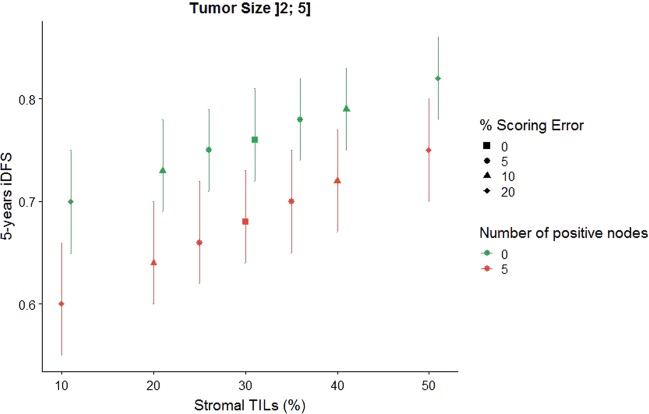
Variation in outcome estimation based on stromal TIL assessment. Shown is the variation in estimated outcome based on sTIL assessment for a 60-year-old patient with a histological grade 3 tumor, 2–5cm in size and receiving anthracycline+taxane based chemotherapy. Presuming a true value for sTILs of 30%, changes in estimated 5-year iDFS for 5, 10, and 20% deviations (increase and decrease) in sTIL assessments are represented with 95% confidence bands. (All calculations were performed using the online triple-negative breast cancer (TNBC)-prognosis tool^9^ available at www.tilsinbreastcancer.org).

The online triple-negative breast cancer (TNBC)-prognosis tool (www.tilsinbreastcancer.org) that contains cumulative data of 9 phase III TNBC-trials^9^, was used to analyze the impact of variation in sTIL assessments (using the sTIL-scores of this analysis) on outcome. The impact on outcome of different sTIL levels is represented in Fig. 9, showing a prototypical example of a 60-year-old patient with a histological grade 3 triple-negative breast carcinoma, measuring between 2 and 5 cm (pT2) and showing 30% sTILs. Assuming she is node negative, if a pathologist properly quantifies the percentage of sTILs, the 5-years invasive disease-free survival (iDFS) is estimated at 76%. If the pathologist deviates down 10% in scoring sTILs (i.e., 20% sTILs), the 5-years iDFS decreases to 73%. Conversely, if the pathologist deviates up 10% in scoring sTILs (i.e., 40% sTILs), the 5-years iDFS goes up to 79%. These differences are modest from a purely prognostic viewpoint, although larger variations would lead to more pronounced differences in outcome estimation. If cutpoints are used to decide on therapy, on the other hand, variation in values around the cut point (as reflected in the concordance rates in Table 1 and Supplemental material) may impact clinical management. Additional examples of outcome estimation as a function of sTILs are provided in the Supplemental material.

### A new resource for pathologists

To assist pathologists in avoiding the different types of pitfalls in the assessment of sTILs identified in this analysis, we have developed an educational tool available via the International Immuno-Oncology Working Group website at www.tilsinbreastcancer.org/pitfalls. Both conventional pictures of microscopic slides and digitized whole slide images (WSIs) of biopsies and surgical resection specimens of breast and other cancers are available to illustrate the described pitfalls. At this point in time, we have included several examples of each of the pitfalls. In the future, we intend to add extra illustrative examples to make this collection a ‘living’ library and continuously evolving learning tool for the pathology community. We invite the pathology community to provide examples of challenging cases for TIL evaluation via the website.

## Discussion

In the current study, we evaluated factors which serve to increase the interobserver variability of manual sTILs assessment. The data were analyzed as both continuous and categorical variables. Despite the challenges pathologists face in scoring sTILs, the reported prognostic and predictive value of sTILs remains consistent across multiple datasets analyzed by independent investigators^9,25^. On the individual patient level, however, we have shown that discrepancies in sTILs scoring between pathologists results in different individual outcome estimations, requiring refinements in the paradigm to maximize benefit and minimize risk.

Notable strengths of this study include the evaluation of both core biopsy and excision specimens, which reflect the reality of clinical practice in which sTIL assessment will be performed. Analyzing the concordance rates across various cutpoints allows us to inform regarding reproducibility to aid in educated cut point selection for future trials. If a singular cutpoint is used, variation in values around that cutpoint can result in misassignment. However, in the setting of an understanding of the scoring error, the cutpoint can be adjusted to a range such that below is X, above is Y and between is indeterminate, and based on a strategy of risk management the overall risk is mitigated. The extensive reference images in this manuscript, as well as the online education resource with further examples (www.tilsinbreastcancer.org/pitfalls), are a valuable reference guide to the pathology community.

A limitation to consider is the poor quality of many of the slides from the excision specimen sections in ring study 3 that were identified as showing the highest discordance. This skewed the evaluation towards technical factors, which are likely to be less of an issue in contemporary clinical practice, but are of relevance in retrospective analyses from older clinical trials. Nonetheless, if presented with such a case in practice, only intact, morphologically assessable areas should be included in sTIL score. If applicable, one could attempt recutting and staining a new slide or selecting a different block for assessment. This information further bolsters the demands for optimal tissue handling and processing.

Among the sources of variability identified, the greatest challenge appears to be dealing with heterogeneous distribution of sTILs. This issue was partially mitigated in ring study 2 which required assessment and averaging of at least 3 separate areas of tumor. The areas were selected by the pathologist to reflect the range of sTIL density and could be within a single core or across separate cores depending on the case. One may postulate that the increased experience of having participated in ring study 1 accounts for the greater concordance in ring study 2; however, the pathologists in ring study 3 had participated in the previous two ring studies and nonetheless showed lower ICC and concordance rates than ring study 2. Ring study 3 was the only study using whole sections compared to core biopsies in the other two studies. One could consider that the increased area of tumor in an excision specimen could lead to increased discordance^26^. In reality, however, many of the core biopsy cases contained multiple tissue cores per slide with multiple separate fragments of tumor, which likely negated any benefit of smaller tumor area. Although the recommendation to score multiple areas and average them in the setting of a heterogeneous tumor is within the published recommendation guidelines^8^, the software in ring study 2 made this a firm requirement. Similarly, use of reference % sTIL images is recommended in the guideline but was a mandatory component of ring study 2. We identified these two key recommendations from the scoring guidelines as having a major impact on consistency of results. These two relatively simple steps: scoring multiple areas in heterogeneous tumors and always using reference images (to minimize personal assessment bias to always “score high” or “score low”)^27^ substantially improve concordance. This re-enforces the central importance of adhering to recommendations in the scoring guidelines. Once factors of heterogeneity are excluded, taking the time to evaluate slides at a sufficiently high power to distinguish lymphocytes from other immune cells as well as mimics can further improve concordance. Being cognizant of lymphoid aggregates around benign ducts and lobules, vessels and DCIS outside of the tumor will help identify these as unrelated to the invasive carcinoma when present within the tumor boundary where these lymphoid aggregates should be excluded from sTIL assessment.

Demonstration of the reproducibility of sTILs scoring is essential for widespread adoption. The importance of sTILs as a biomarker is being increasingly recognized resulting in recommendations by multiple respected groups. The 2019 St. Gallen Panel recommended that sTILs be routinely characterized in TNBC for their prognostic value^8,15^. As of yet, however, insufficient data exists to recommend sTILs as a test to guide systemic treatment. In addition, the latest iteration of the *WHO Classification of Breast* *Tumours* also includes information on sTILs^28^.

Stromal TIL-assessment by pathologists is now recognized as an analytically and clinically validated biomarker. There is Level 1B evidence that high levels of sTILs are associated with improved outcome and an enhanced response to neoadjuvant therapy in triple-negative and HER2-positive breast cancers^7,11–14,29^, and are prognostic for disease-free and overall survival in early triple-negative breast cancers treated with standard anthracycline-based adjuvant chemotherapy^4,6,9^. Clinical utility [likelihood of improved outcomes from use of the biomarker test compared to not using the test]^30^ remains to be defined. A recent retrospective study demonstrated that patients with Stage I TNBC with >30% sTILs had excellent survival outcomes (5-year overall survival rate of 98% [95%CI: 95% to 100%]) in the absence of chemotherapy^31^, paving the way for future randomized trials of chemotherapy de-escalation in early TNBC.

Clinical utility for sTILs is also likely to come from cancer immunotherapy, a rapidly emerging field aimed at augmenting the power of a patient’s own immune system to recognize and destroy cancer cells. The immune system is able to impart selective pressure on cancer cells resulting in immune-evading clones. Stromal TILs can identify tumors amenable to immunotherapies targeting immunosuppression^32^. Checkpoint inhibitors of programmed cell death protein 1 (PD-1) and programmed death-ligand 1 (PD-L1) are promising therapeutic interventions, however predicting tumor response to these agents remains challenging^33^. There is increasing hesitation about the utility of the current predictive biomarker PD-L1 expression by IHC. The utility of PD-L1 IHC is undermined by the well-characterized geographic and temporal heterogeneity and dynamic expression on tumor or tumor-infiltrating immune cells^34^. Technical differences, variable expression and variation in screening thresholds for PD-L1 expression across assays pose additional limitations. Studies have shown that although pathologists can score PD-L1 on tumor cells with high concordance, even with training they are not concordant in scoring PD-L1 on immune cells^35–37^. There are emerging data that sTILs, as assessed by the consensus-method defined by the TIL Working Group, are predictive for response to checkpoint-inhibition in metastatic triple-negative and HER2-positive breast cancer^38,39^. The response rate is linear with increasing sTILs related to a higher response rate^39^. Further investigations are ongoing.

As we look to the future, automated sTIL assessment holds the promise of adding complementarity to the current pathological evaluation of breast cancers. A heterogeneous pattern of lymphocyte infiltration may be better addressed with computational pathology methods^40,41^. Further, there is some evidence that the spatial distribution of TILs may provide additional prognostic information^42^. One study reported improved prognosis and response to chemotherapy in TNBC with a diffuse, homogeneous lymphocyte distribution versus a heterogeneous distribution^43^. This requires further evaluation. Lymphocytes are particularly well-suited to image analysis, as it is easier to recognize these small blue dark cells against a stromal background than, for example, to distinguishing malignant cells from normal epithelium. There is a surge in the development of machine learning methods for TIL assessment^44^. The histopathologic diagnostic responsibility will continue to reside with the pathologist. Image analysis and computation pathology, which are proven to be faster and more reproducible, are adjuncts that aid the pathologist but do not replace the function of histopathologic interpretation. Until these tools are available, the well-educated and well-trained pathologist is the best approach. Rigorous training, evaluation and practice are well documented to result in improved intra- and inter-pathologist reproducibility. It is hoped that by highlighting the specific pitfalls in sTIL assessment in this manuscript – the forewarned pathologist is the forearmed pathologist. Ongoing efforts to ensure reliable and reproducible reporting of sTILs are a key step in their smooth progression into the routine clinical management of breast cancer.

## Methods

### Identification of cases demonstrating variability using ring studies by the TIL-Working Group

We identified 3 ring studies evaluating concordance of sTIL assessment in breast cancer performed by TIL-WG pathologists, for which we could obtain individual pathologist data and images^22,23^. The ring studies were performed on clinical trials material. All participating patients gave written informed consent to sample collection and the use of these samples for translational biomarker research, as approved by the Ethics Commission of the Charité Universitätsmedizin Berlin. All relevant ethical regulations have been complied with for this study. In ring study 1, 32 pathologists evaluated 60 scanned breast cancer core biopsy slides^22^. Scores were missing for 5 slides; the missing values were replaced by the mean of the 31 remaining scores. Ring study 2 was an extension of the first study. A subset of 28 of the original 32 pathologists participated and scored 60 different scanned breast cancer core biopsy slides^22^. Ring study 3 was performed by six TIL-WG pathologists who independently scored 100 scanned whole slide breast cancer cases^23^. In total, 220 slides were included. For each individual slide, the variability (standard deviation) among pathologists was measured from individual sTILs scores. The slides with the highest 10% standard deviation were identified for evaluation.

### Statistical analysis of scoring variance between pathologists

The R software environment was used for statistical computing and graphics (version 3.5.0). Scoring variance among pathologists was analyzed using the Intraclass Correlation Coefficient (ICC). ICC estimates and their 95% confidence intervals were calculated based on individual-pathologist rating (rather than average of pathologists), absolute-agreement (i.e., if different pathologists assign the same score to the same patient), 2-way random-effects model (i.e., both pathologists and patients are treated as random samples from their respective populations)^45^. To compute ICC, we used the “aov” function to fit the data with a two-way random effect ANOVA model (readers and cases). We followed Fleiss and Shrout’s method to approximate the ICC confidence intervals^46^. We created custom code for the concordance analysis. Concordance rates for all pairs of pathologists were calculated at several sTIL density cutpoints: <1 vs ≥1%; <5 vs ≥5%; <10 vs ≥10%; <30 vs ≥30%; <75 vs ≥75%. Specifically, each concordance was the percent agreement from the 2 × 2 table created from each cutpoint and pair of readers. The analyses were performed and confirmed independently by two separate groups (RE & SM; Gustave Roussy) and (BDG & WC; FDA). Details of the concordance analysis are presented in Supplementary Tables 1–3.

### Evaluation of sources of variability in the three-ring studies

Slides for ring study 1 and 2 were Whole Slide Images (WSI) and were viewed using a virtual microscope program (CognitionMaster Professional Suite; VMscope GmbH). Each slide identified as showing the top 10% discordance, as well as specifically chosen cases (1 outlier low sTIL case in ring study 1 and 3 additional high discordance cases from ring study 3) were examined in order to identify potential confounding factors for routine sTIL assessment.

### Clinical significance of variability in sTIL assessment by pathologists

The impact of variation in sTILs on outcome estimation was evaluated using the online triple-negative breast cancer (TNBC)-prognosis tool (www.tilsinbreastcancer.org) that contains cumulative data of 9 phase III TNBC-trials. The sTIL scores of this analysis were used as the ground truth. Specifically, different patient profiles were defined based on standard clinicopathological factors: age, tumor size, number of positive nodes, tumor histological grade and treatment. For a specific patient profile and a value of sTIL, the tool was used to calculate the 5-year invasive disease-free survival (iDFS). The iDFS is defined as the date of first invasive recurrence, or second primary or death from any cause.

### Reporting summary

Further information on research design is available in the Nature Research Reporting Summary linked to this article.

## Supplementary information

Supplementary files

Reporting Summary Checklist

## Data Availability

The histology images supporting Fig. 2 and Figs. 4–8, are publicly available in the figshare repository, as part of this record: 10.6084/m9.figshare.11907768^47^. Data supporting Fig. 3, Tables 1–5 and Supplementary Tables 1–3 are not publicly available in order to protect patient privacy. These datasets can be accessed on request from Dr. Roberto Salgado, upon the completion of a Data Usage Agreement, according to policies from the German Breast Group and NSABP, as described in the data record above. Figure 9 and supplementary figures 1–8, were generated using the publicly available prognosis tool at www.tilsinbreastcancer.org/, which utilises datasets from a pooled analysis of 9 phase 3 breast cancer trials, including BIG 02-98, ECOG 1199, ECOG 2197, FinHER, GR, IBCSG 22-00, IEO, PACS01 and PACS04 (10.1200/JCO.18.01010). This paper is intended to serve as a practical reference for practicing pathologists.

## Code availability

The code is available from the corresponding author by request.
