# Supplementary material for: Pitfalls in assessing stromal tumor infiltrating lymphocytes (sTILs) in breast cancer
Source: NPJ Breast Cancer. 2020 May 12;6:17. doi: 10.1038/s41523-020-0156-0 (PMC7217863; doi:10.1038/s41523-020-0156-0)
Supplement: Supplementary file 1 — Supplementary files [file 41523_2020_156_MOESM1_ESM.pdf]

Outcome estimation in different clinicopathologic scenarios across variable sTIL values

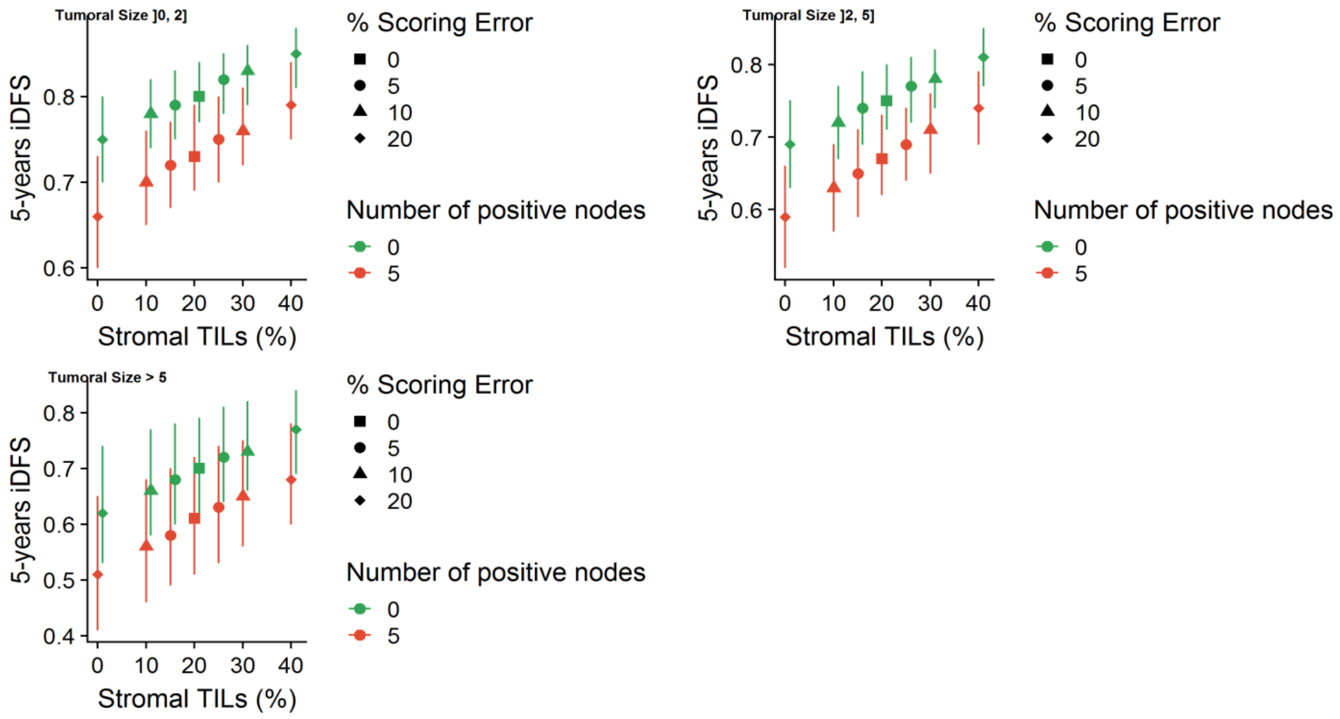

Supplementary Figure 1. Age 50, histological grade 1 or 2, sTILs = 20%, anthracycline + taxane treatment

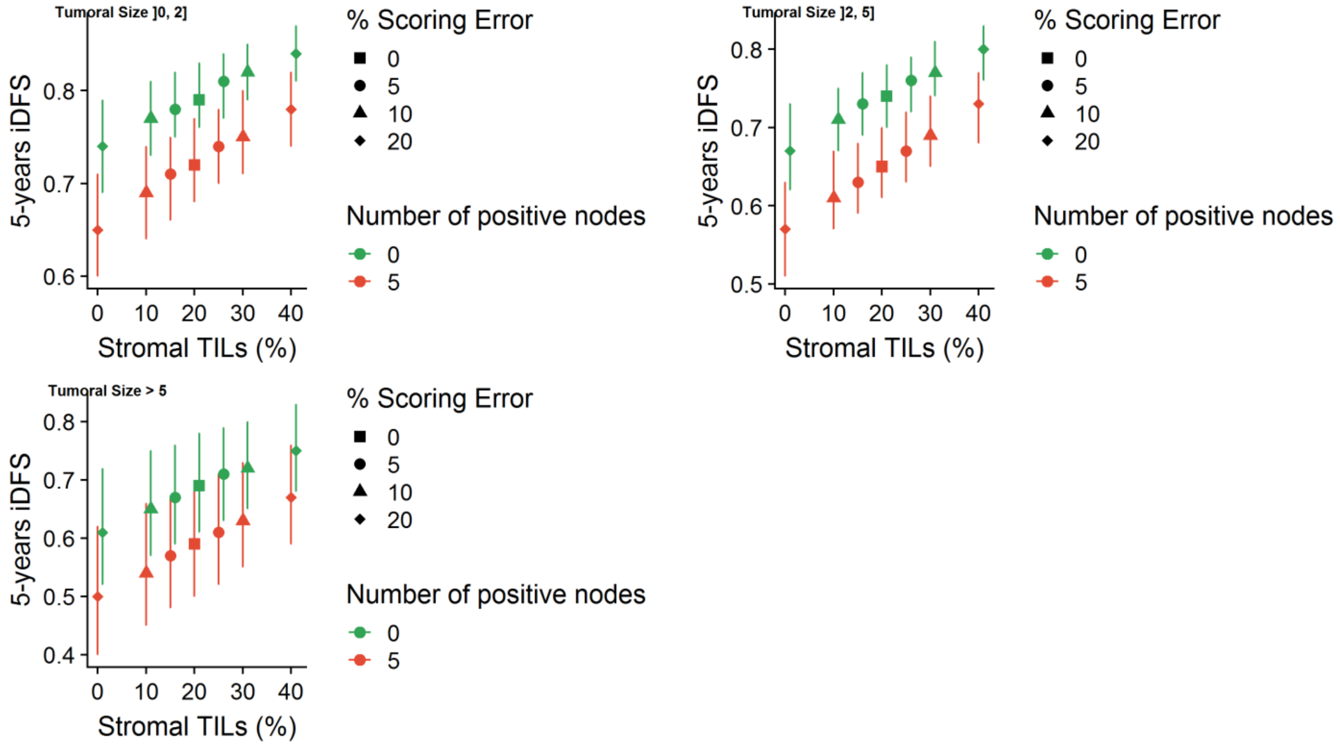

Supplementary Figure 2. Age 50, histological grade 3, sTILs = 20%, anthracycline + taxane treatment

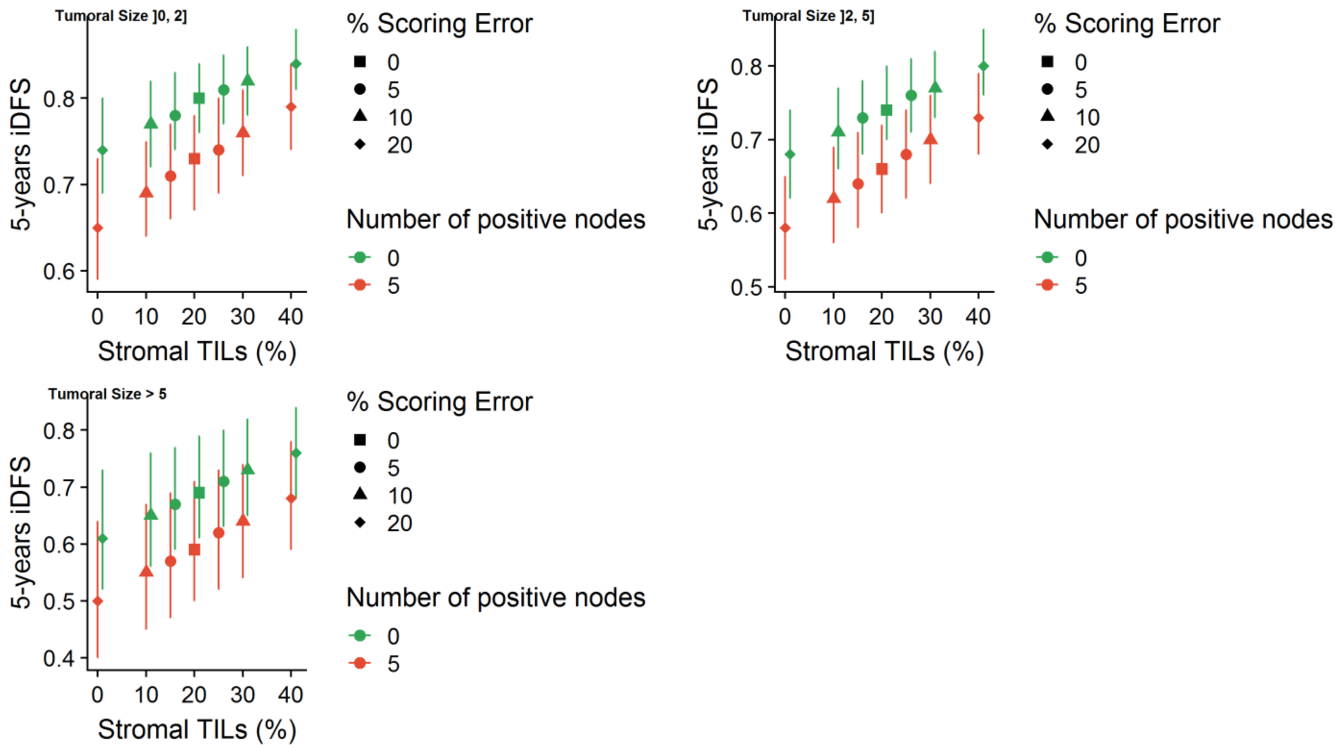

Supplementary Figure 3. Age 60, histological grade 1 or 2, sTILs = 20%, anthracycline + taxane treatment

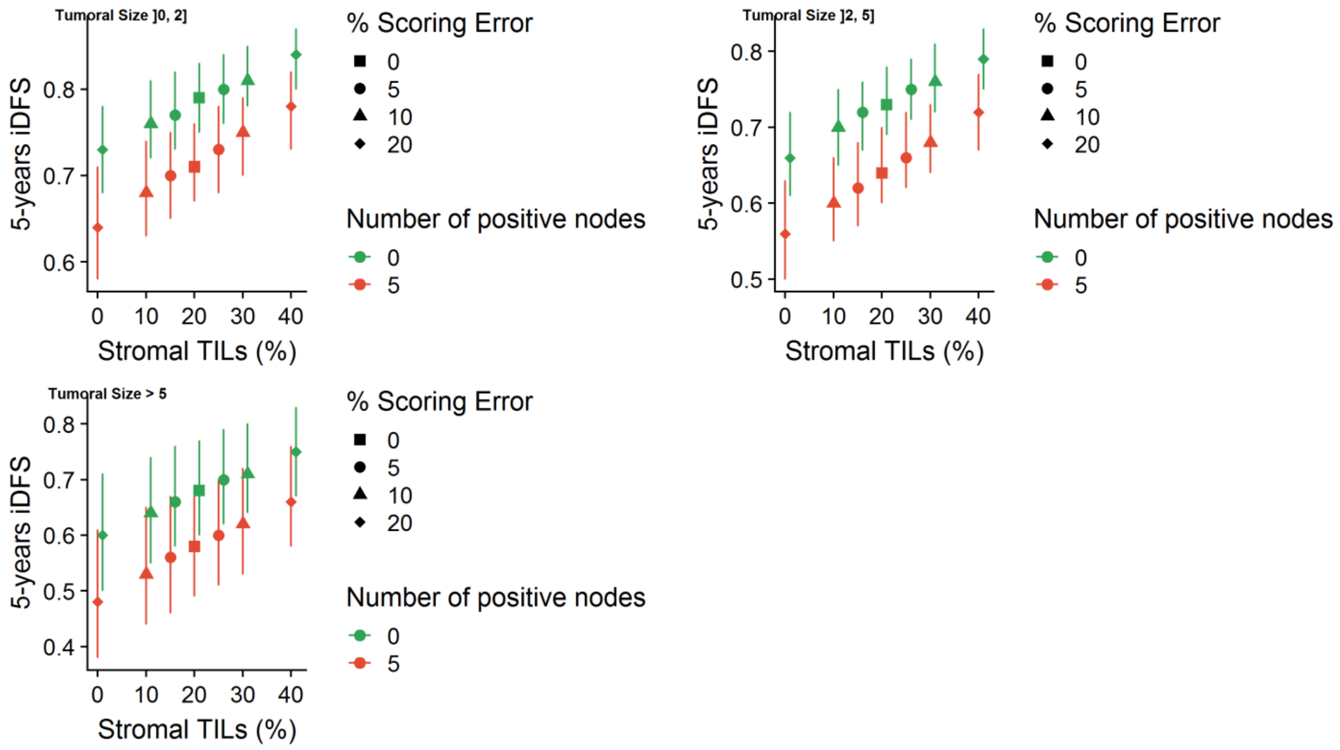

Supplementary Figure 4. Age 60, histological grade 3, sTILs =20%, anthracycline + taxane treatment

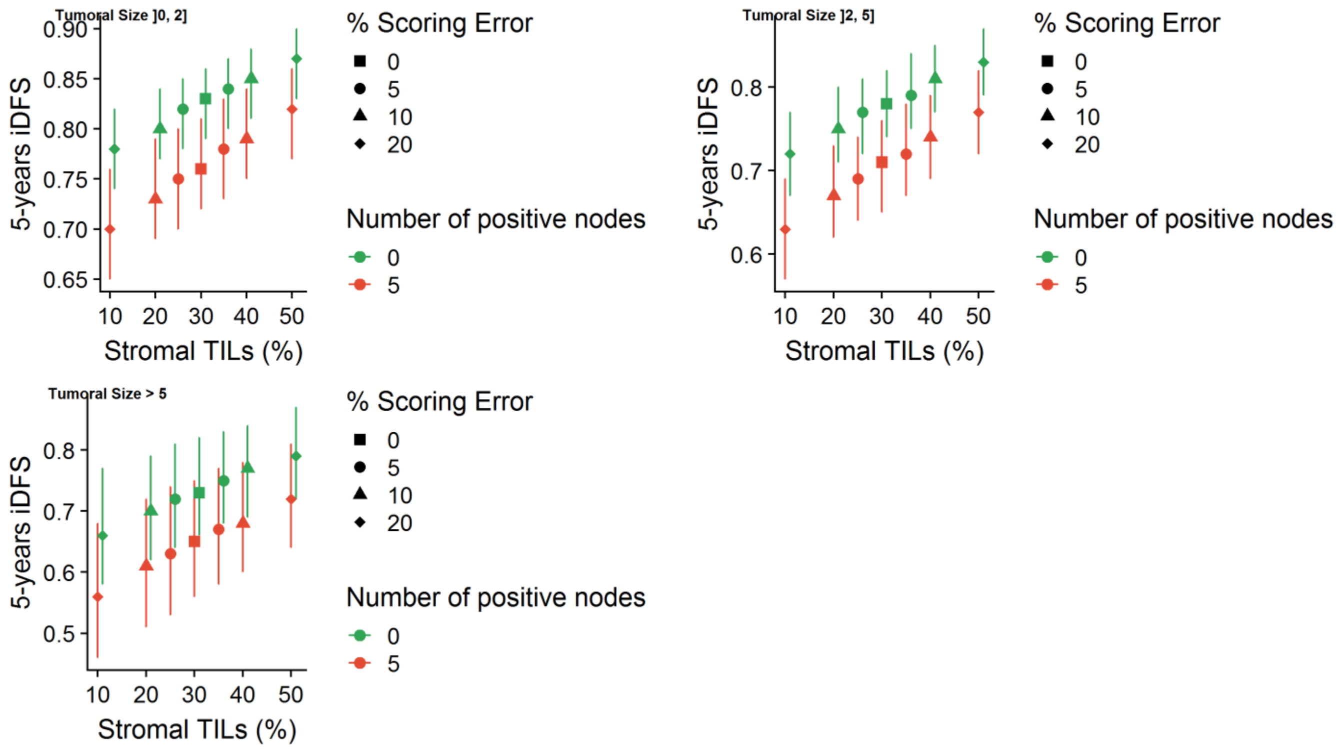

Supplementary Figure 5. 50, histological grade 1 or 2, sTILs = 30%, anthracycline + taxane treatment

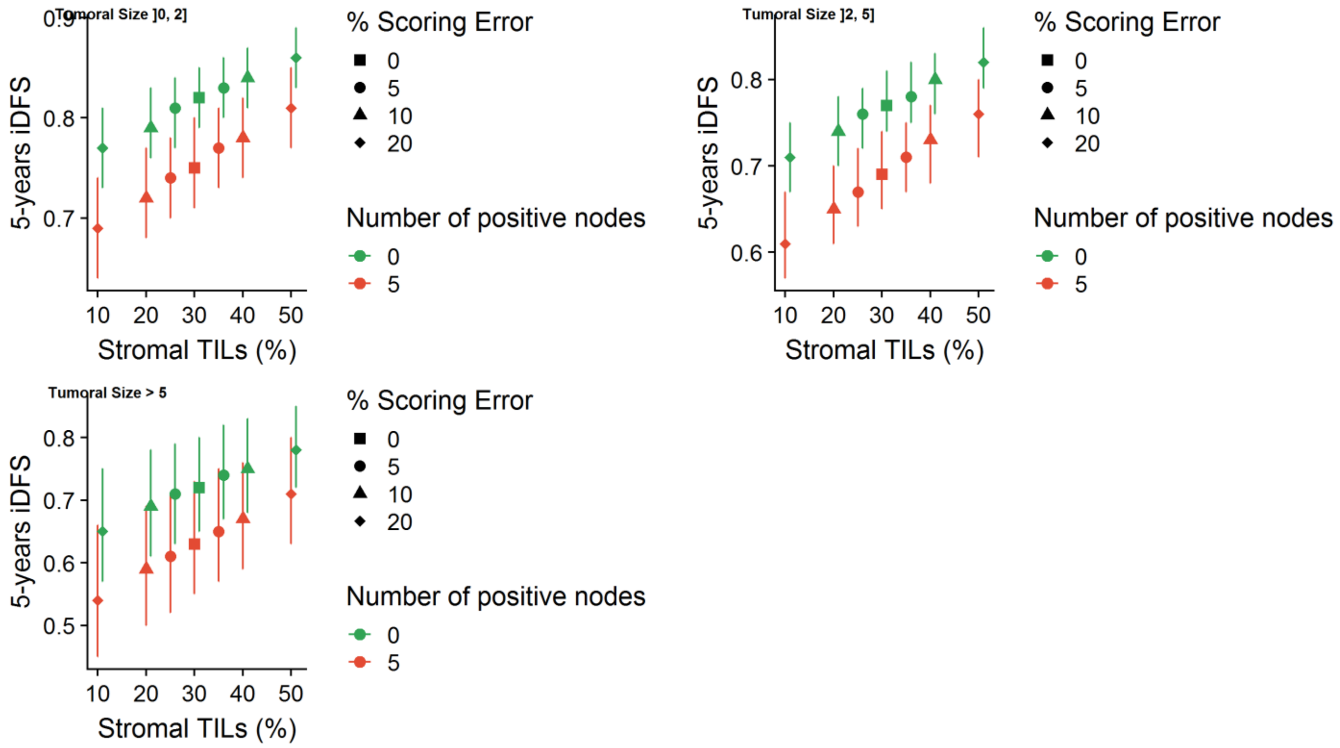

Supplementary Figure 6. Age 50, histological grade 3, sTILs = 30%, anthracycline + taxane treatment

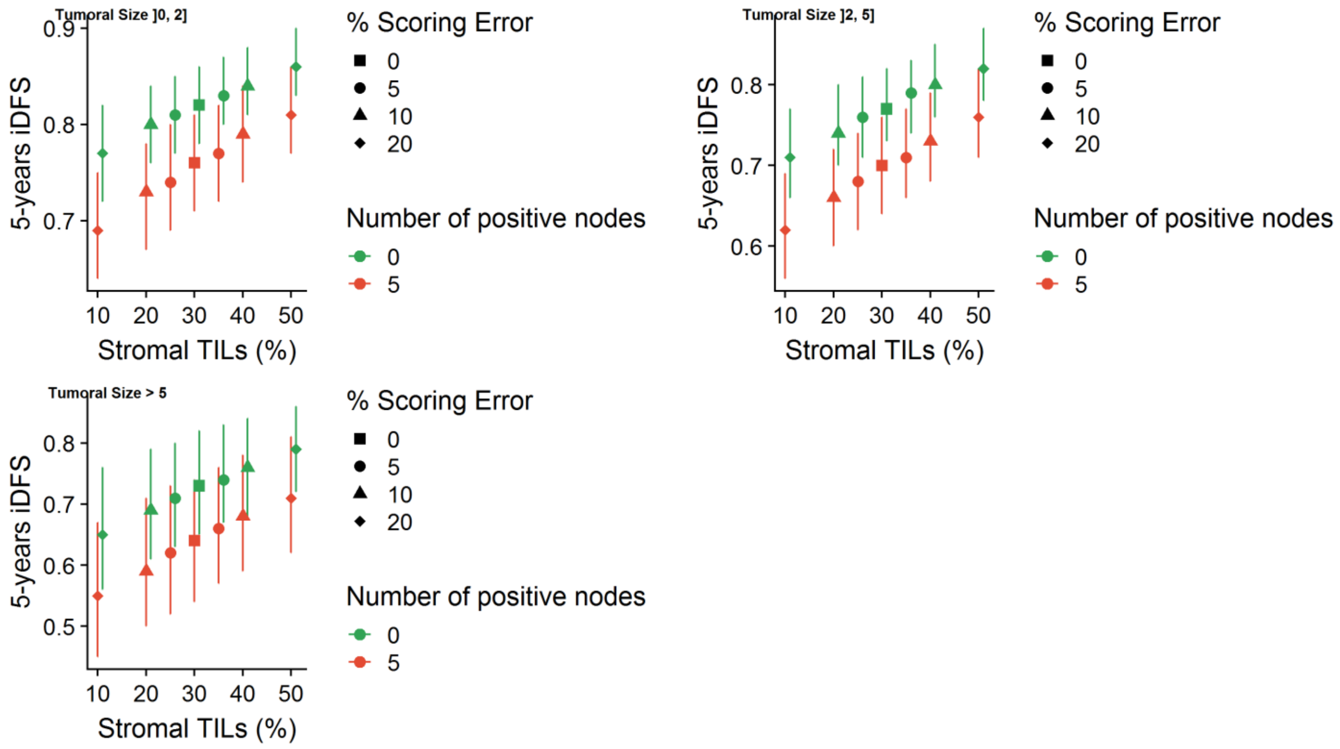

Supplementary Figure 7. Age 60, histological grade 1 or 2, sTILs = 30%, anthracycline + taxane treatment

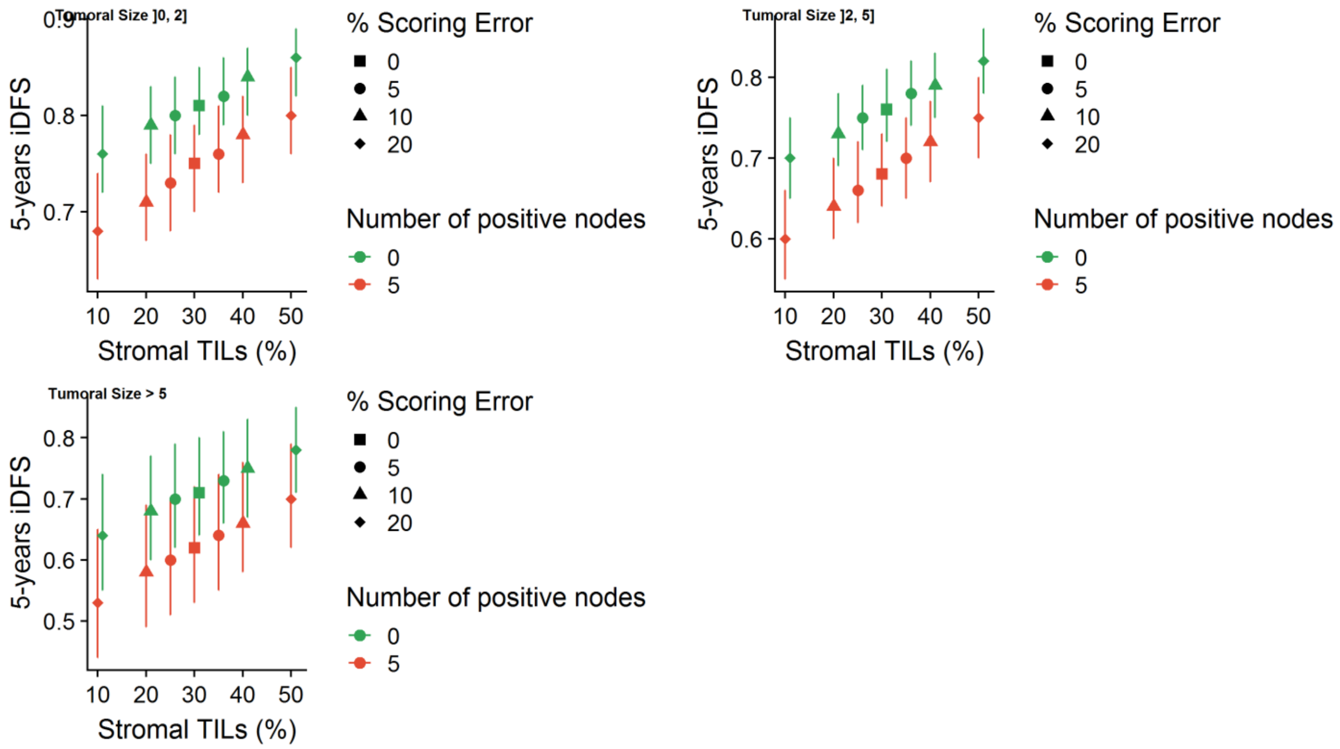

Supplementary Figure 8. Age 60, histological grade 3, sTILs =30%, anthracycline + taxane treatment

Concordance among pathologists

Supplementary Table 1. Assessment of concordance per pathologist by comparing with the gold standard in the first dataset (RS1) by replacing missing data by the mean (32 pathologists who scored 60 slides)

Pathologist N°	ICC single measures	Concordance rate for different cut points				
		1%	5%	10%	30%	75%
**1**	0,84 [0,74; 0,9]	1,00	0,90	0,83	0,90	0,90
**2**	0,83 [0,69; 0,9]	1,00	0,87	0,78	0,85	0,98
**3**	0,86 [0,73; 0,92]	1,00	0,97	0,88	0,92	0,95
**4**	0,83 [0,28; 0,94]	1,00	0,88	0,80	0,77	0,92
**5**	0,88 [0,37; 0,96]	1,00	0,92	0,87	0,90	0,92
**6**	0,81 [0,7; 0,88]	1,00	0,93	0,83	0,90	0,97
**7**	0,9 [0,83; 0,94]	1,00	0,88	0,87	0,87	0,95
**8**	0,86 [0,74; 0,92]	0,02	0,88	0,83	0,90	0,97
**9**	0,79 [0,02; 0,93]	1,00	0,90	0,82	0,75	0,90
**10**	0,86 [0,75; 0,92]	0,10	0,92	0,82	0,95	0,92
**11**	0,92 [0,85; 0,96]	0,18	0,77	0,82	0,92	0,95
**12**	0,82 [0,36; 0,93]	1,00	0,77	0,72	0,83	0,95
**13**	0,92 [0,68; 0,97]	0,30	0,73	0,78	0,92	0,97
**14**	0,64 [0,12; 0,84]	0,03	0,95	0,88	0,73	0,75
**15**	0,9 [0,81; 0,95]	1,00	0,90	0,90	0,93	0,93
**16**	0,81 [0,7; 0,88]	1,00	0,88	0,92	0,88	1,00
**17**	0,9 [0,82; 0,94]	1,00	0,87	0,85	0,93	0,93
**18**	0,83 [0,71; 0,9]	0,07	0,92	0,88	0,92	0,87
**19**	0,76 [0,63; 0,85]	1,00	0,83	0,75	0,82	0,97
**20**	0,91 [0,67; 0,96]	1,00	0,92	0,87	0,90	0,92
**21**	0,84 [0,75; 0,9]	1,00	0,83	0,83	0,88	0,93
**22**	0,85 [0,72; 0,91]	1,00	0,97	0,87	0,87	0,97
**23**	0,91 [0,79; 0,96]	0,10	0,88	0,87	0,93	0,95
**24**	0,82 [0,55; 0,92]	0,18	0,92	0,85	0,87	0,95
**25**	0,76 [0,57; 0,86]	1,00	0,87	0,88	0,80	0,92
**26**	0,93 [0,78; 0,97]	0,02	0,88	0,87	0,90	0,95
**27**	0,88 [0,73; 0,94]	0,08	0,72	0,73	0,87	0,95
**28**	0,71 [0,49; 0,83]	1,00	0,83	0,88	0,85	0,82
**29**	0,89 [0,82; 0,94]	1,00	0,87	0,80	0,93	0,97
**30**	0,9 [0,75; 0,95]	1,00	0,88	0,83	0,83	0,98
**31**	0,84 [0,75; 0,9]	1,00	0,92	0,95	0,95	0,93
**32**	0,56 [0,13; 0,77]	1,00	0,92	0,77	0,73	0,97

Supplementary Table 2. Assessment of concordance per pathologist by comparing with the gold standard in the second dataset (RS2) (28 pathologists who scored 60 slides)

Pathologist N°	ICC single measures	Concordance rate for different cut points				
		1%	5%	10%	30%	75%
1	0,96 [0,92; 0,98]	0,95	0,90	0,85	0,95	0,93
2	0,97 [0,94; 0,98]	0,97	0,97	0,90	0,98	0,95
3	0,95 [0,92; 0,97]	0,97	0,93	0,92	0,95	0,98
4	0,92 [0,84; 0,96]	0,93	0,87	0,97	0,95	0,95
5	0,97 [0,92; 0,99]	0,97	0,95	0,85	0,95	0,97
6	0,93 [0,87; 0,96]	1,00	0,93	0,88	0,97	0,92
7	0,94 [0,79; 0,97]	0,97	0,95	0,93	0,95	0,93
8	0,97 [0,94; 0,98]	0,90	0,83	0,88	0,95	0,97
9	0,94 [0,9; 0,96]	0,92	0,82	0,88	0,95	0,92
10	0,97 [0,94; 0,98]	0,05	0,92	0,95	0,97	0,93
11	0,96 [0,93; 0,98]	0,95	0,97	0,90	0,97	0,97
12	0,96 [0,89; 0,98]	0,95	0,83	0,90	0,95	1,00
13	0,97 [0,95; 0,98]	0,97	0,95	0,90	1,00	0,92
14	0,93 [0,86; 0,96]	0,95	0,98	0,97	0,92	0,97
15	0,97 [0,94; 0,98]	0,98	0,92	0,90	0,98	0,93
16	0,95 [0,92; 0,97]	0,97	0,95	0,90	0,97	0,95
17	0,92 [0,83; 0,96]	0,05	0,93	0,87	0,92	0,92
18	0,92 [0,81; 0,96]	0,97	0,97	0,90	0,93	0,87
19	0,92 [0,86; 0,95]	0,92	0,82	0,92	1,00	0,88
20	0,89 [0,78; 0,94]	0,93	0,93	0,93	0,92	0,97
21	0,96 [0,93; 0,97]	0,97	0,97	0,97	0,95	0,90
22	0,97 [0,95; 0,98]	0,97	0,92	0,92	0,93	0,98
23	0,95 [0,89; 0,97]	0,93	0,92	0,88	0,97	0,95
24	0,9 [0,83; 0,94]	1,00	0,95	0,87	0,93	0,90
25	0,97 [0,95; 0,98]	0,05	0,95	0,87	0,98	0,98
26	0,95 [0,84; 0,98]	0,97	0,98	0,95	0,95	0,92
27	0,92 [0,64; 0,97]	0,05	1,00	0,90	0,93	0,97
28	0,95 [0,85; 0,97]	0,92	0,80	0,87	0,92	0,98

Supplementary Table 3. Assessment of concordance per pathologist by comparing with the gold standard in the third dataset (RS3) (6 pathologists who scored 100 full slides)

Pathologist N°	ICC single measures	Concordance rate for different cut points				
		**1%**	**5%**	**10%**	**30%**	**75%**
**1**	0,95 [0,93; 0,97]	0,97	0,96	0,88	0,93	0,98
**2**	0,94 [0,91; 0,96]	0,02	0,96	0,90	0,95	0,98
**3**	0,94 [0,9; 0,96]	0,89	0,84	0,85	0,95	0,97
**4**	0,9 [0,84; 0,94]	0,02	0,05	0,81	0,90	0,98
**5**	0,72 [0,52; 0,83]	0,88	0,75	0,83	0,88	0,98
**6**	0,84 [0,74; 0,9]	0,02	0,97	0,93	0,90	0,91
